# RPS27L Enhances Myogenesis and Muscle Mass by Targeting IGF1 Through Liquid‐Liquid Phase Separation

**DOI:** 10.1002/advs.202512354

**Published:** 2025-08-31

**Authors:** Xiaoqin Liu, Yilong Yao, Junyu Yan, Mu Zeng, Xinhao Fan, Yijie Tang, Jiju Li, Yanwen Liu, Shanying Yan, Wei Wang, Lijuan Chen, Ruipu Chen, Yuxin Huang, Honor Calnan, Heng Wang, Graham Gardner, Yalan Yang, Zhonglin Tang

**Affiliations:** ^1^ Shenzhen Branch Guangdong Laboratory of Lingnan Modern Agriculture Key Laboratory of Livestock and Poultry Multi‐omics of MARA Agricultural Genomics Institute at Shenzhen Chinese Academy of Agricultural Sciences Shenzhen 518124 China; ^2^ Kunpeng Institute of Modern Agriculture at Foshan Agricultural Genomics Institute Chinese Academy of Agricultural Sciences Foshan 528226 China; ^3^ College of Environmental and Life Sciences Murdoch University Murdoch WA 6150 Australia; ^4^ College of Animal Science Shandong Provincial Key Laboratory for Livestock Germplasm Innovation & Utilization Shandong Agricultural University Taian 271000 China

**Keywords:** IGF1, liquid‐liquid phase separation, muscle mass, myofiber sizes, RPS27L, skeletal muscle

## Abstract

RNA‐binding proteins (RBPs) play a pivotal role in post‐transcriptional regulation of gene expression, critically influencing skeletal myogenesis, muscle growth, and regeneration. Despite the recent identification of RBP *Rps27l* (ribosomal protein S27‐like) as a regulator affecting myogenic proliferation and differentiation, its functions and regulatory mechanisms in skeletal muscle development remain largely unknown. In this study, it is observed that muscle‐specific *Rps27l* knock‐in (M─KI) mice exhibit significantly increased muscle mass, enlarged myofiber size, a higher proportion of fast‐twitch myofibers, and enhanced muscle regeneration capabilities compared to wild‐type controls. Overexpression of Rps27l promotes myoblast proliferation while inhibiting differentiation in skeletal muscle cells. Mechanistically, it is revealed that the expression of *Rps27l* is negatively regulated by SIX4, a myogenic transcription factor. The N‐terminal intrinsically disordered region of RPS27L facilitates liquid‐liquid phase separation (LLPS) and interacts with IGF1 to collaboratively regulate myogenesis. The findings uncover the novel regulatory roles of RPS27L in skeletal muscle and highlight the significance of RPS27L‐driven LLPS in myogenesis.

## Graphical abstract

Muscle‐specific RPS27L knock‐in mice exhibit increased body weight, muscle mass, and enhanced muscle regenerative capacity. RPS27L drives myogenesis by promoting myoblast proliferation, while inhibiting myogenic differentiation. Mechanistically, RPS27L targets IGF1 protein by liquid‐liquid phase separation through its N‐terminal intrinsically disordered region, to up‐regulate IGF1 expression and collaboratively regulate myogenesis.

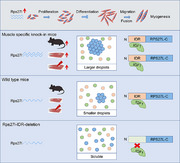

## 1 Introduction

Skeletal muscle is the largest tissue component in humans and animals, serving as the primary tissue responsible for locomotion,^[^ ^1^ ^]^ and engages in cross‐talk with other organs and tissues,^[^ ^2^ ^]^ such as adipose tissue, the brain, and the intestine.^[^ ^3^ ^]^ The development of skeletal muscle initiates with a population of cells in the mesoderm somite and progresses through a series of highly orchestrated myogenesis processes, culminating in the formation of multinucleated myotubes and ultimately mature myofibers.^[^ ^4^, ^5^ ^]^ Upon injury, skeletal muscle regeneration commences with the activation of skeletal muscle satellite cells (MuSCs) and recapitulates embryonic myogenesis, during which muscle‐specific genes are reactivated.^[^ ^6^, ^7^ ^]^ Numerous transcription factors (TFs) and non‐coding RNAs (ncRNAs) have been identified to play central roles in these processes, such as paired box 3 (PAX3),^[^ ^8^ ^]^ myogenin,^[^ ^9^ ^]^ special AT‐rich sequence‐binding protein 2 (SATB2),^[^ ^10^ ^]^ and circular RNA‐circFgfr2.^[^ ^11^ ^]^

RNA‐binding proteins (RBPs) are pivotal in orchestrating gene expression at both transcriptional and post‐transcriptional levels.^[^ ^12^ ^]^ By interacting with target RNAs, RBPs form ribonucleoprotein complexes that play crucial roles in transcription, RNA processing, degradation, and nuclear export, ultimately fine‐tuning gene expression dynamics.^[^ ^13^ ^]^ Numerous RBPs are specifically expressed in muscle and are essential for skeletal muscle development.^[^ ^14^, ^15^ ^]^ For instance, RNA binding motif protein 24 (RBM24) is initially localized in the cytoplasm of myoblasts and later translocated to the nucleus at differentiated myotubes,^[^ ^16^, ^17^ ^]^ as well as muscle regeneration.^[^ ^18^ ^]^ Muscle blind‐like 1 (MBNL1) promotes myoblast differentiation through alternative splicing (AS), while its family member, muscle blind‐like 3 (MBNL3), inhibits this process.^[^ ^19^, ^20^ ^]^ The lack of one allele of TAR DNA binding protein (TDP43) results in the formation of smaller myofibers during muscle regeneration.^[^ ^21^ ^]^

Liquid‐liquid phase separation (LLPS) is an emerging mechanism for explaining the precise spatial‐temporal regulation of cellular biological processes. It compartmentalizes proteins and nucleic acids into micron‐scale, liquid‐like, membrane‐less bodies with specific functions known as biomolecular droplets,^[^ ^22^ ^]^ offering new insights into skeletal muscle development.^[^ ^23^ ^]^ For example, the muscle‐specific TF BTB‐zinc finger protein Tono instructs *Drosophila* muscle development through LLPS.^[^ ^24^ ^]^ The transcriptional coactivator with PDZ‐binding motif (TAZ) exhibits phase separation properties and represses myogenesis.^[^ ^25^ ^]^ Additionally, excess cellular prion protein (PrPC) inhibits skeletal muscle cell differentiation via miRNA‐enhanced LLPS.^[^ ^26^ ^]^ Recent studies highlighted the intriguing ability of RBPs to undergo LLPS through intrinsically disordered regions (IDRs).^[^ ^27^, ^28^ ^]^

Our recent study revealed ribosomal protein S‐27 like (RPS27L) regulates skeletal myogenesis and is associated with meat production in pigs,^[^ ^29^ ^]^ expanding beyond its recognized roles in apoptosis,^[^ ^30^ ^]^ and neoplasia.^[^ ^31^ ^]^ To further investigate the functions and regulatory mechanism of RPS27L in skeletal muscle development and growth, we generated muscle‐specific *Rps27l* knock‐in (M─KI) mice, given that homozygous Rps27l knockout in mice leads to early postnatal lethality.^[^ ^30^ ^]^ We found that RPS27L functions as a positive regulator of skeletal muscle growth and regeneration. During myogenesis, RPS27L promotes myoblasts proliferation while inhibiting myogenic differentiation. Notably, we highlighted the regulation of SIX4/RPS27L/IGF1 axis in myogenesis through LLPS. Our findings uncovered previously unrecognized roles of RPS27L in skeletal muscle growth and regeneration, suggesting its potential as a therapeutic target for muscular diseases in humans and a candidate gene for the improvement of meat production traits in livestock.

## 2 Results

### 2.1 Muscle‐Specific Rps27l Knock‐in Mice Demonstrate Increased Muscle Mass and Myofiber **Size**

**Figure 1:**
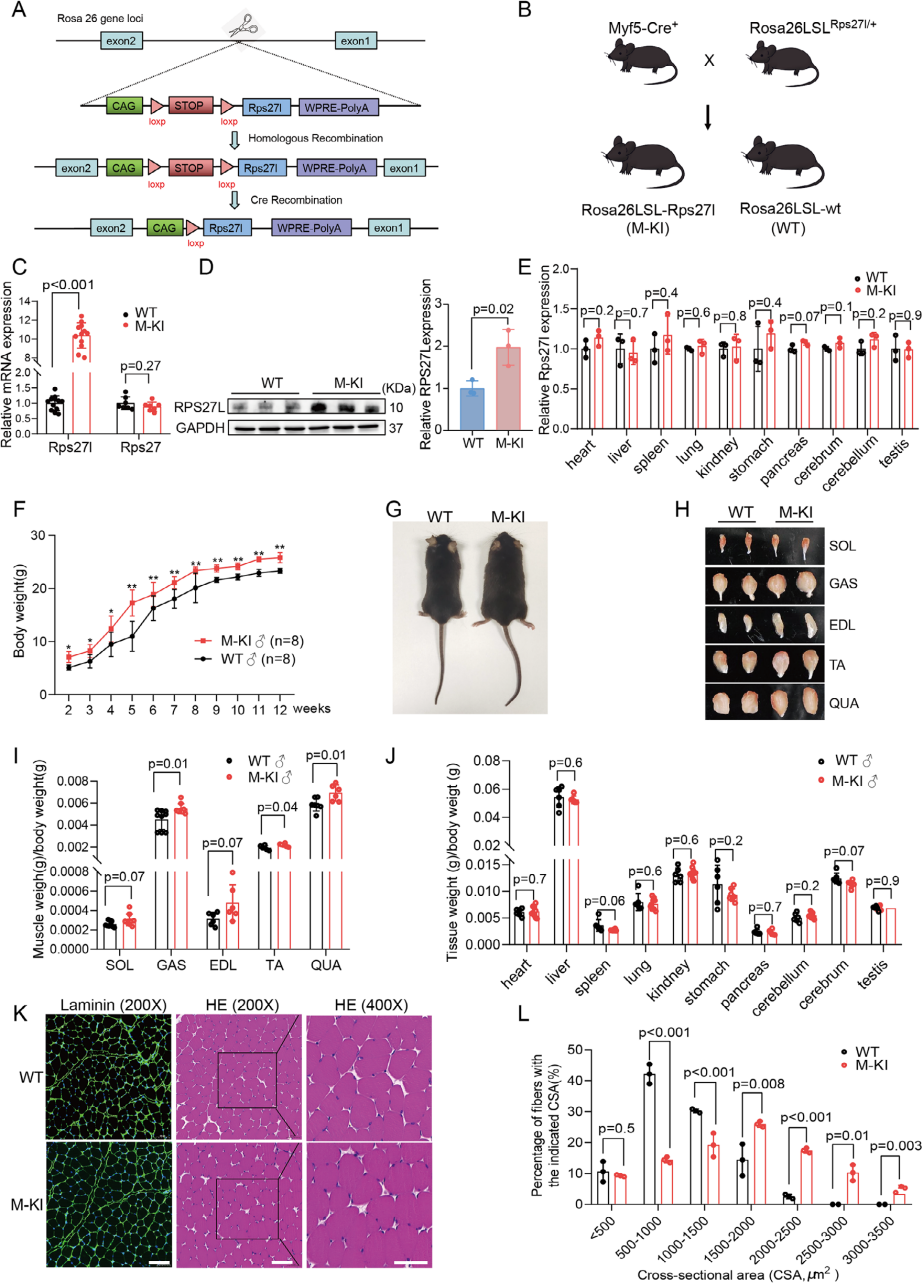
Rps27l knock‐in mice exhibit heavier muscle mass and larger myofiber size. A) Schematic diagram of *Rps27l* conditional knock‐in mice generated using CRISPR/CAS9 technology at Rosa 26 gene loci. B) Breeding strategies to generate muscle‐specific *Rps27l* knock‐in (M─KI) mice using Myf5‐Cre mice. C,D) Expression analysis of *Rps27l* mRNA by RT‐qPCR (**C**) and protein by western blot (**D**) in GAS muscles from 2‐month‐old mice. E) *Rps27l* mRNA expression in non‐muscle tissues/organs of 2‐month‐old M─KI and their corresponding negative siblings (WT) analyzed by RT‐qPCR, n=3 per group. F) Growth curve of male M─KI and WT mice from 2 to 12 weeks of age, n=8 per group. G,H) Representative morphology of adult male mice (**G**) with five typical muscles (**H**). QUA, quadriceps femoris muscle; GAS, gastrocnemius muscle; SOL, soleus muscle; TA, tibialis anterior muscle; EDL, extensor digitorum longus muscle. I,J) Ratio of five typical skeletal muscles (**I**) and non‐muscle tissues/organs (**J**) to body weight (n ≥6 per group) in adult male mice. K) Representative laminin immunofluorescence (left) and H&E‐stained (200× and 400×) images of QUA muscles from 2‐month‐old mice. The scale bars represent 100 µm. L) Quantitative analysis of myofiber size in 2‐month‐old mice revealed the right shift distribution in M─KI compared to WT controls. Data are presented as mean ± SEM. Exact *P* values are shown, ^*^ *P* < 0.05, ^**^ *P* < 0.01, and ^***^ *P* < 0.001. Unpaired two‐tailed Student's *t*‐test (**C**, **D**, **E**, **I**, **J**, **and** **L**) and two‐way ANOVA (**F**).

We employed conditional knock‐in (KI) C57BL/6J mice targeting exons 1 and 2 of the *Rps27l* gene at Rosa26 loci using CRISPR/Cas9 technology. RosaLSL^Rps27l/Rps27l^ mice were generated through homologous recombination (**Figure** **1**A). Utilizing the Cre‐loxP recombination system involving Myf5‐Cre and RosaLSL^Rps27l/Rps27l^ mice, we generated muscle‐specific *Rps27l* KI mice (Rosa26LSL^Rps27l/Rps27l^; Myf5‐cre^+^, abbreviated as M─KI) and corresponding negative controls (Rosa26LSL^Rps27l/Rps27l^; Myf5‐cre^–^, abbreviated as WT) (Methods; Figure 1B; Figure S1A, Supporting Information). We evaluated the knock‐in efficiency and confirmed Rps27l was significantly overexpressed in M─KI skeletal muscles at both mRNA (Figure 1C) and protein (Figure 1D) levels compared to WT controls, while the expression of its homolog ***Rps27*** remained unchanged (Figure 1C). Additionally, no expression differences of *Rps27l* mRNA were observed in other non‐muscle tissues/organs between these two genotypes (Figure 1E). These results indicated that we successfully generated muscle‐specific RPS27L KI mice.

Both male and female M─KI mice exhibited a significant increase in body weight and body size compared to their WT siblings starting from 2 weeks of age (Figure 1F,G; Figure S1B, Supporting Information). Comparing with WT mice, the quadriceps femoris (QUA), gastrocnemius (GAS), soleus (SOL), tibialis anterior (TA), and extensor digitorum longus (EDL) muscles were much heavier in both male and female M─KI mice at 4 months of age (Figure 1H,I; Figure S1C, Supporting Information). As expected, the weights of non‐muscle tissues/organs showed no significant changes (Figure 1J; Figure S1D, Supporting Information). We next performed hematoxylin‐eosin (HE) and immunofluorescence staining assays to measure cross‐sectional areas (CSAs) of skeletal muscles. Results showed that larger myofibers were detected in both QUA (Figure 1K) and GAS (Figure S1E, Supporting Information) muscles of M─KI mice. Analyses of myofiber size distribution demonstrated that M─KI mice exhibited a higher proportion of larger myofibers (> 1500 µm^2^, Figure 1L; Figure S1F, Supporting Information). Together, these results revealed that RPS27L overexpression led to increased myofiber sizes, muscle mass, and body weight in mice.

### 2.2 *Rps27l* Knock‐in Mice Exhibit an Increase Proportion of Fast‐Twitch Myofibers and Enhanced Muscle Regeneration Capacity

**Figure 2:**
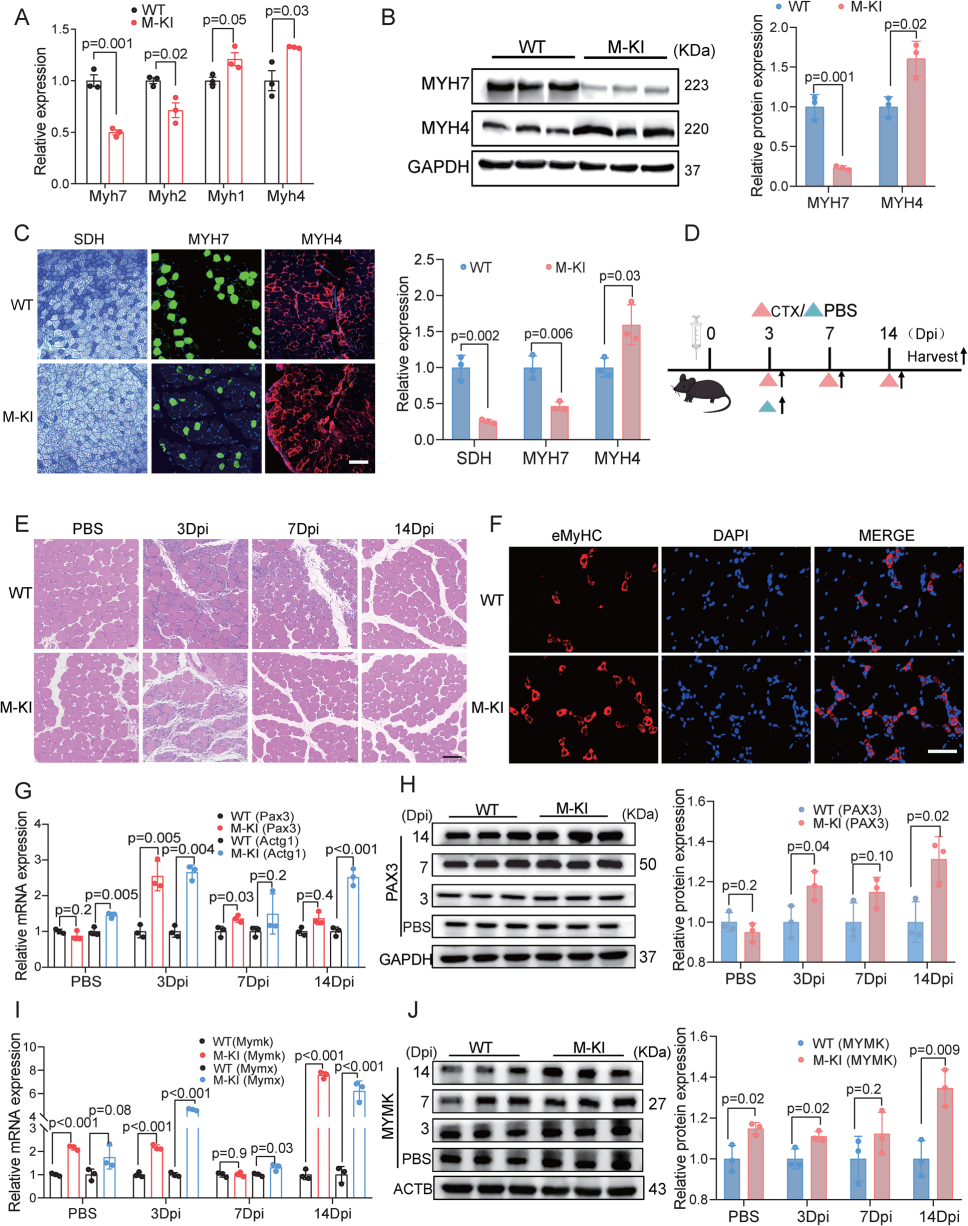
M─KI mice exhibit an increase in glycolytic myofibers and enhanced muscle regeneration capability. A) Quantitative RT‐qPCR analysis of myofiber marker gene expression in QUA muscles from 2‐month‐old mice. B) Western blot analysis of MYH4 and MYH7 protein expression in QUA muscles from 2‐month‐old mice. C) Representative images and analysis of SDH, MYH7, and MYH4 staining in QUA muscles from 2‐month‐old mice, DAPI staining (blue) for cell nuclei. The scale bars represent 100 µm. D) Scheme timeline of drug administration and sample collection in the CTX‐injured regeneration model of GAS muscles from 2‐month‐old mice, n=3 per group. E) Representative H&E‐stained sections of CTX‐injured muscle regeneration at multiple time points in M─KI and WT mice. The scale bars represent 100 µm. F) Representative immunofluorescence images of eMyHC (red) in CTX‐injured muscles at 14 Dpi. The scale bars represent 100 µm. G,H) Quantitative analysis of migration markers in CTX‐injured regenerating muscles at both mRNA (RT‐qPCR, **G**) and protein (Western blot, **H**) levels. I,J) Quantitative analysis of fusion markers in CTX‐injured regenerating muscles at both mRNA (RT‐qPCR, **I**) and protein (Western blot, **J**) levels. Data are presented as mean ± SEM. Exact P values are shown. Unpaired two‐tailed Student's *t*‐test (**A, B, C, G, H, I,** and **J**).

Skeletal muscles consist of diverse myofiber types with variations in size and metabolic characteristics.^[^ ^32^ ^]^ We primarily investigated the expression of MYH7 (a marker of slow‐twitch oxidative, type I fibers) and MYH4 (a marker of fast glycolytic, type II fibers) in QUA muscles from M─KI and WT mice. Results revealed a significant upregulation of MYH4 and a substantial downregulation of MYH7 in M─KI mice at both mRNA (**Figure** **2**A) and protein (Figure 2B) levels. Correspondingly, both succinic dehydrogenase (SDH) staining and immunostaining of MYH7 and MYH4 supported a shift toward a higher prevalence of fast glycolytic myofibers and a decreased presence of slow‐twitch oxidative myofibers in M─KI mice (Figure 2C). This observation prompted us to examine whether RPS27L overexpression affects glucose metabolism in mice, given the association between elevated glycolytic metabolism and potential improvements in glucose homeostasis.^[^ ^33^, ^34^ ^]^ We conducted intraperitoneal glucose‐ and insulin‐tolerance tests (IP‐GTT and IP‐ITT). Compared to their WT siblings, M─KI mice exhibited reduced glucose tolerance and slower insulin‐stimulated glucose clearance (Figure S2A,B, Supporting Information). These findings highlighted the role of RPS27L in altering muscle fiber‐types and the maintenance of glucose homeostasis.

We further explored the effects of RPS27L on muscle regeneration. CTX injections were administered to induce damage on GAS muscles in 2‐month‐old M─KI and WT mice. Injured GAS muscles were collected at 3, 7, and 14 days of post‐injection (Dpi, Figure 2D). Histological analysis of H&E staining revealed fewer centrally nucleated cells in M─KI mice from 7 Dpi onward, and in WT mice from 14 Dpi onward, demonstrating the enhanced skeletal muscle repair capability in M─KI mice (Figure 2E). Immunostaining of eMyHC, a hallmark of muscle regeneration, also demonstrated a higher number of regenerated myofibers in M─KI mice (Figure 2F). Moreover, marker genes related to proliferation (Figure S2C, Supporting Information), differentiation (Figure S2D, Supporting Information), migration (Figure 2G,H), and fusion (Figure 2I,J) exhibited up‐regulated expression during muscle regeneration in M─KI mice. Collectively, these results suggest that overexpression of RPS27L enhances muscle regeneration capacity.

### 2.3 RPS27L Promotes Myoblast Proliferation While Repressing Myogenic Differentiation

**Figure 3:**
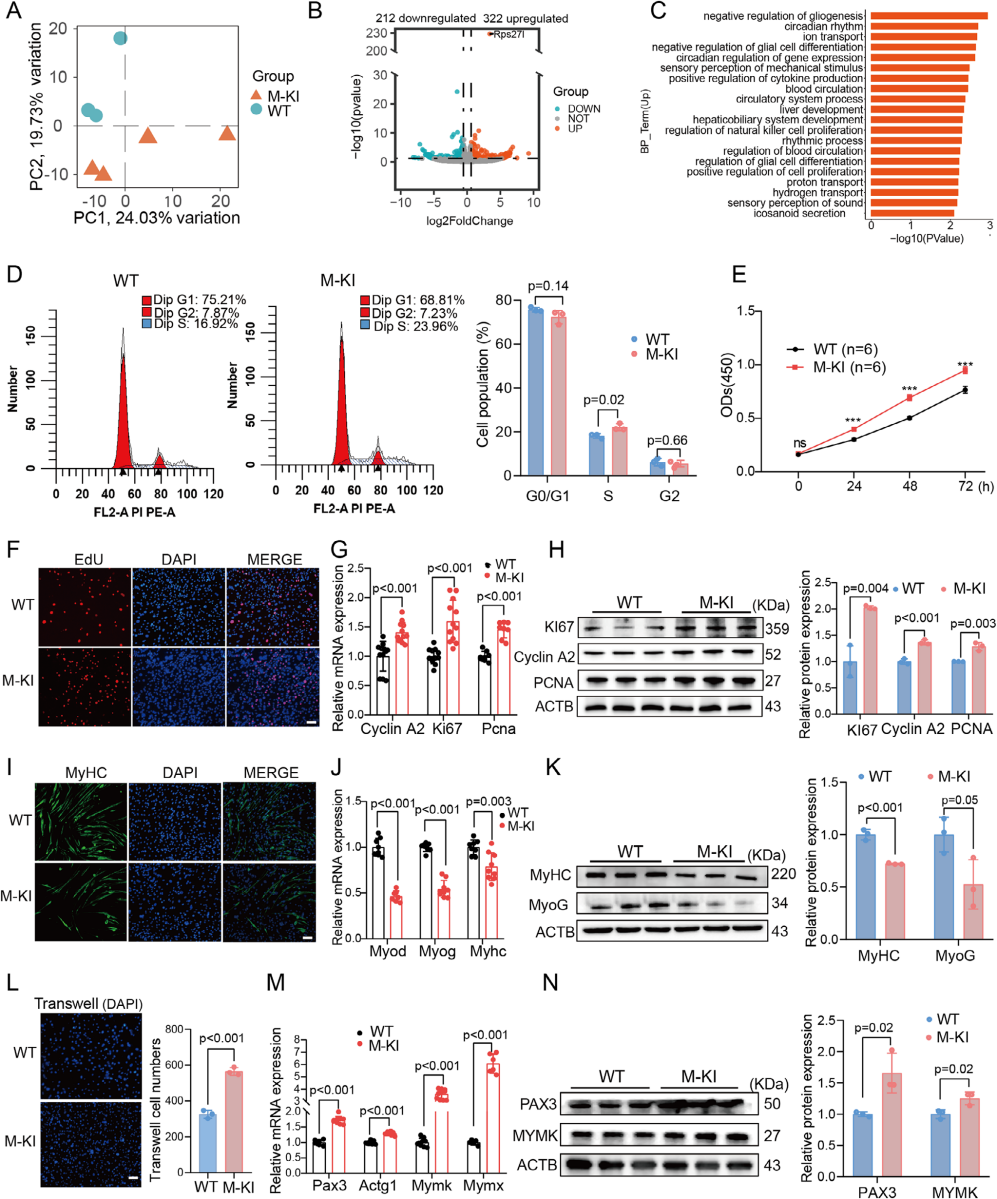
Rps27l promotes myoblast proliferation and represses differentiation in MuSCs. A) PCA plot of RNA‐seq data from QUA muscles from 2‐month‐old M─KI and WT mice. B) The volcano plot showing gene expression changes in GAS muscles comparing M─KI versus WT mice (red: up‐regulated; blue: down‐regulated). C) GO enrichment analysis of up‐regulated genes in M─KI mice. D) Cell cycle analysis of MuSCs from M─KI and WT mice by flow cytometry. E–H) Proliferation capacity of MuSCs from M─KI and WT mice was evaluated using CCK‐8 (**E**) and EdU assays (**F**), alongside RT‐qPCR (**G**) and Western blotting (**H**) to quantify proliferation marker expression at mRNA and protein levels, respectively. I–K) The differentiation capacity of MuSCs from M─KI and WT mice was assessed by immunofluorescence for MyHC (**I**), with complementary quantification of differentiation markers via RT‐qPCR (**J**) and Western blotting (**K**). L) Representative images of migrated MuSCs from M─KI and WT mice via transwell assays, with DAPI staining (blue) showing cell nuclei. M,N) Expression of migration‐ and fusion‐related markers in MuSCs from M─KI and WT mice was analyzed by RT‐qPCR (**M**) and Western blotting (**N**). The scale bars represent 100 µm. Data are presented as mean ± SEM. Exact P values are shown, ^*^ *P* < 0.05, ^**^ *P* < 0.01, and ^***^ *P* < 0.001. Unpaired two‐tailed Student's *t*‐test (**D**, **G**, **H**, **J**, **K**, **M,** and **N**) and two‐way ANOVA (**E**).

To investigate the functions of RPS27L in myogenesis, we conducted RNA‐seq analysis on dissected QUA muscles from 2‐month‐old M─KI mice and their WT siblings. Principal Component Analysis (PCA) revealed distinct separation between these two groups, suggesting global gene expression differences between M─KI and WT mice (**Figure** **3**A). A total of 534 differentially expressed genes (DEGs) were identified, with 322 up‐ and 212 down‐regulated genes in M─KI mice compared to WT mice (Figure 3B; Table S1, Supporting Information). GO analysis suggested significant enrichment of up‐regulated genes related to cell proliferation and differentiation (Figure 3C).

We isolated MuSCs from the QUA muscles of M─KI and WT mice. The cell cycle assay indicated that M─KI MuSCs reduced the number of cells entering the G0/G1 phase and increased the number of cells entering the S phase (Figure 3D). Both the CCK‐8 assay (Figure 3E) and ethynyl‐2′‐deoxyuridine (EdU) incorporation assay (Figure 3F) showed higher proliferation activity in M─KI MuSCs. The expression of proliferation markers (Pcna, Ki67, and Cyclin A2) was significantly enhanced at both mRNA (Figure 3G) and protein (Figure 3H) levels in M─KI MuSCs. Additionally, immunostaining assays indicated a significant reduction in the number of myotubes in M─KI MuSCs (Figure 3I), consistent with the repressed expression of myogenic markers such as MyoD, MyoG, and MyHC (Figure 3J,K). In addition, we carried out overexpression and knockdown experiments of RPS27L in C2C12 myoblasts using a pcDNA3.1 vector and a small interfering RNA (siRNA), respectively. Results confirmed that RPS27L overexpression led to an increase in myoblast proliferation activity (Figure S3A–F, Supporting Information) and a decrease in differentiation ability (Figure S3G–I, Supporting Information). The opposite effect on myogenesis was observed under RPS27L knockdown (Figure S3J–R, Supporting Information).

Meanwhile, the transwell assay demonstrated that more cells were collected in M─KI MuSCs compared to WT MuSCs, exhibiting an enhanced migration capability when *Rps27l* was knocked in (Figure 3L). The markers for migration and fusion were up‐regulated in M─KI MuSCs compared to WT MuSCs (Figure 3M,N). Collectively, these findings indicated that RPS27L promoted myoblast proliferation, migration, fusion, and repressed myogenic differentiation in skeletal muscle cells.

### 2.4 RPS27L is Negatively Regulated by Transcription Factor SIX4

**Figure 4:**
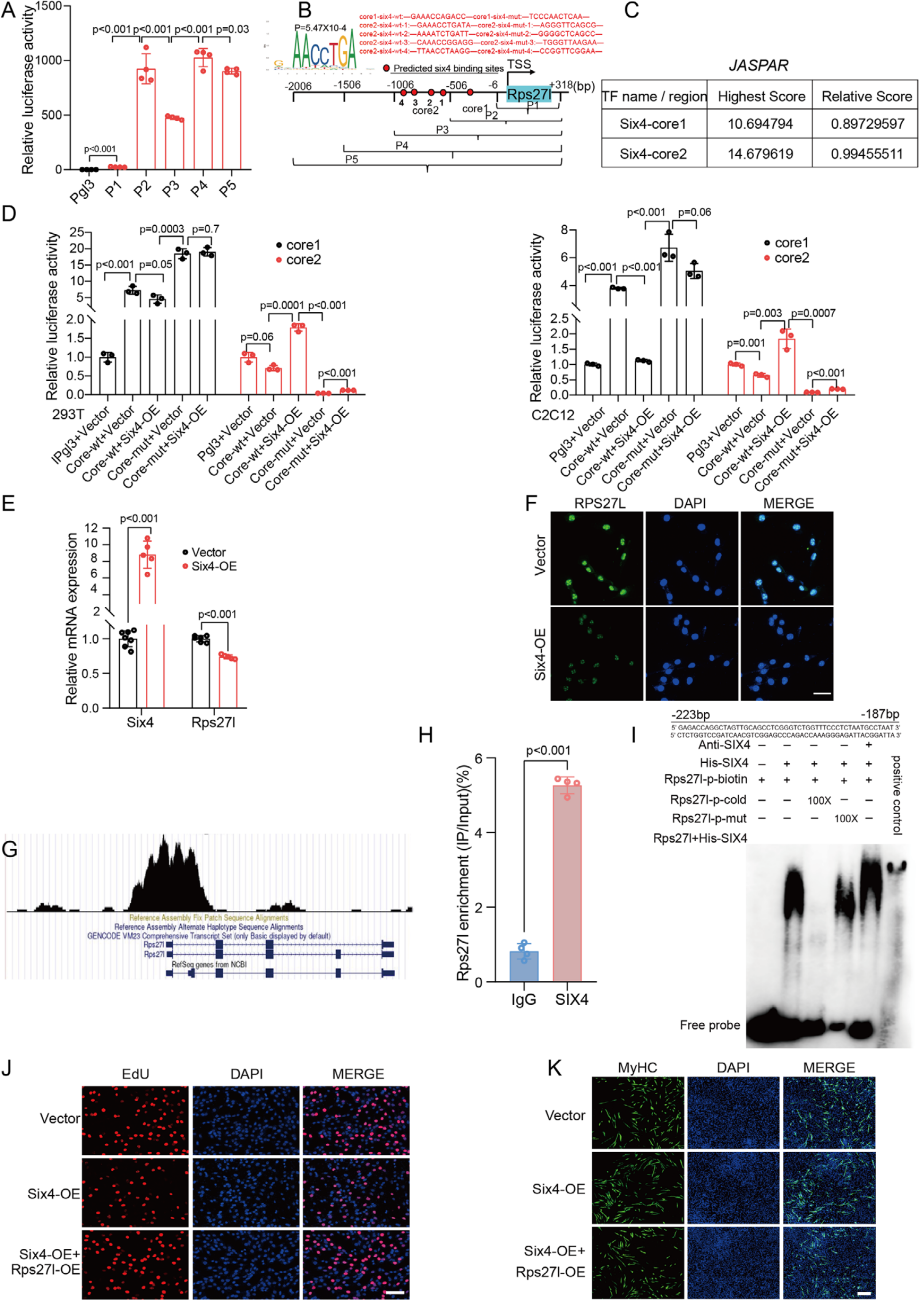
SIX4 binds to *Rps27l* promoter and negatively regulates its expression. A) Dual luciferase assays **were performed** to identify the core promoter regions of *Rps27l* in HEK293T cells. B) Schematic illustration of predicted SIX4 binding sites and their corresponding sequences in the *Rps27l* promoter region. C) Binding scores for SIX4 in core promoter regions, as predicted by JASPAR (http://jaspar.genereg.net) analysis. D) Dual luciferase reporter assays demonstrate SIX4 binding activity on the two core regions of *Rps27l* promoter in both HEK293T cells (left) and C2C12 myoblasts (right). E,F) SIX4‐mediated negative regulation of *Rps27l* was demonstrated at both transcriptional (RT‐qPCR; **E**) and protein (immunofluorescence; **F**) levels. G) A significant SIX4 ChIP‐seq peak was identified in the *Rps27l* promoter region. The ChIP‐seq dataset for C2C12 myoblasts was obtained from the Citrome DB (http://cistrome.org/db/#/). H,I) SIX4 binding to the *Rps27l* promoter was validated in M─KI MuSCs by ChIP‐qPCR (**H**) and further confirmed through in vitro EMSA assay (**I**). J) The reduced proliferation ability by Six4 overexpression was rescued by Rps27l overexpression as evidenced by EdU incorporation assay. K) Rps27l overexpression abolished the enhanced differentiation capacity of Six4 overexpression by immunofluorescence staining. Data are presented as mean ± SEM. Exact P values are shown. Unpaired two‐tailed Student's *t*‐test (**A**, **D, E,** and **H**). [Correction added on 30 September 2025 after online publication: The fluorescence images in Figure 4K and the western blot in Figure 5C were updated. Also, Figure S5G is also updated.]

To identify the upstream regulator of RPS27L, we first attempted to pinpoint its core promoter region. We divided the *Rps27l* promoter into five consecutive sections (P1–P5) and constructed corresponding luciferase reporters for each section. The dual luciferase assay indicated the presence of two core promoter regions (core1 and core2) (**Figure** **4**A). Next, we used JASPAR program (http://jaspar.genereg.net) to predict TFs that could potentially bind to the identified core promoter regions. Five putative binding sites of SIX4 (Figure 4B), an known regulator of muscle regeneration,^[^ ^35^ ^]^ were found in the core regions with high confident scores (Figure 4C). The luciferase assay revealed that SIX4 overexpression suppressed the relative activity in core1 but enhanced the activity in core2 in both HEK293T and C2C12 cells. When all the binding sites in core1 and core2 were separately mutated, the dual‐luciferase activity was significantly restored, regardless of whether an empty vector or Six4 overexpression construct was co‐transfected (Figure 4D). Collectively, these results demonstrate that Six4 exerts its regulatory function through directly binding to these binding sites.

Notably, Six4‐driven luciferase activity was significantly stronger in core1 than core2 in both HEK293T and C2C12 myoblasts (Figure 4D). This suggests that any potential transcriptional activation mediated by core2 may be offset by repression through core1. To verify this, a luciferase reporter containing the *Rps27l* promoter (P5, upstream 2 kb of TSS) was constructed and subjected to dual luciferase assay in C2C12 myoblasts. As anticipated, overexpression of SIX4 decreased the promoter activity of *Rps27l* in C2C12 myoblasts (Figure S4A, Supporting Information). Additionally, we confirmed that SIX4 overexpression negatively regulates RPS27L at both mRNA (Figure 4E) and protein (Figure 4F) levels in C2C12 myoblasts. Based on publicly available SIX4 ChIP‐seq data in C2C12 myoblasts from Cistrome DB (http://cistrome.org/db/#/), a peak was also observed in the *Rps27l* promoter (Figure 4G).^[^ ^35^ ^]^

To further validate the binding in vivo, chromatin immunoprecipitation (ChIP) was performed using SIX4‐specific antibody, followed by qPCR with primers flanking the primary functional binding site in M─KI MuSCs. Results showed that *Rps27l* promoter region containing the primary functional binding site was enriched by 5.3‐fold (Figure 4H). Electrophoretic mobility shift assay (EMSA) was next conducted to further confirm their binding in vitro. After obtaining the purified SIX4 fusion proteins, three types of probes were designed to target the primary functional binding site: the *Rps27l* biotin‐labeled probe, a cold competition probe, and a mutant probe. EMSA results showed that SIX4 was detected when using the *Rps27l* biotin‐labeled probe. However, the signal either disappeared or decreased, respectively, with the addition of the cold competition or mutant probe prior to adding the *Rps27l* biotin‐labeled probe (Figure 4I; Figure S4B, Supporting Information). Collectively, these findings demonstrate that SIX4 directly binds to the *Rps27l* promoter and represses its expression.

To determine whether *Rps27l* overexpression could rescue the functional effects of *Six4* overexpression, we performed complementary rescue experiments in C2C12 myoblasts. Results demonstrated that *Rps27l* overexpression effectively counteracted the anti‐proliferative effects of *Six4* overexpression. This restoration was evidenced by EdU incorporation assay (Figure 4J), accompanying with the upregulated expression of proliferation markers at both mRNA (Figure S4C, Supporting Information) and protein levels (Figure S4D, Supporting Information). Conversely, *Rps27l* overexpression counteracted Six4‐induced myotube promotion (Figure 4K), and expression of *Myog* and *Myhc* (Figure S4E,F, Supporting Information). These rescue results establish that SXI4 and RPS27L function as opposing regulators in myogenesis, with SIX4 negatively regulating RPS27L expression.

### 2.5 RPS27L Directly Interacts with IGF1 in Myogenesis

**Figure 5:**
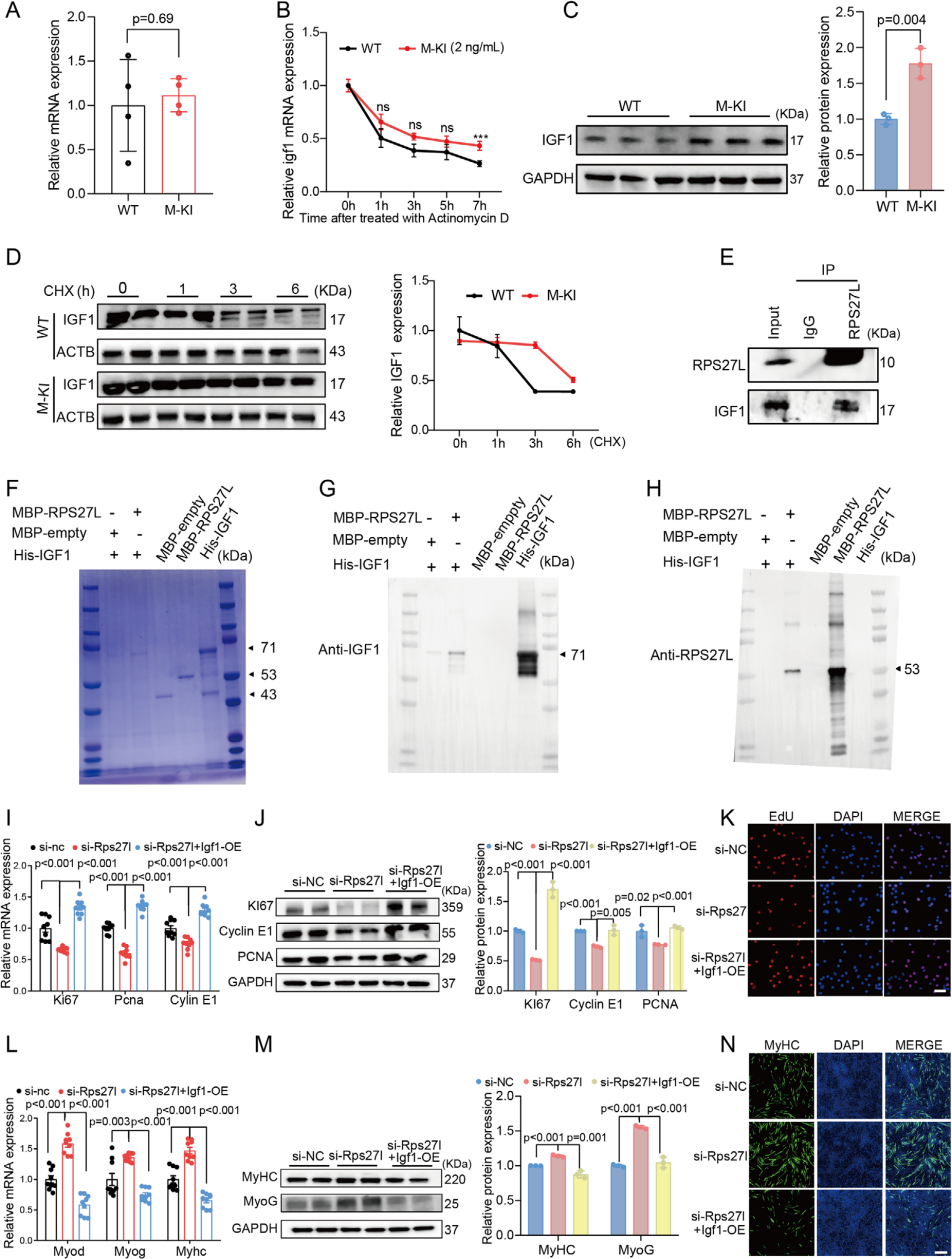
RPS27L directly binds to IGF1, co‐regulating myogenesis. A) Relative *Igf1* mRNA expression levels in GAS muscle were compared between M─KI and WT mice, with RNA‐seq data validated by RT‐qPCR. B) *Rps27l* mRNA stability in MuSCs was assessed following actinomycin D treatment (2 ng mL^−1^). C) IGF1 protein expression levels in muscles from 2‐month‐old M─KI and WT mice were analyzed by Western blot. D) The half‐life of IGF1 protein in MuSCs from M─KI and WT mice was determined by cycloheximide (CHX) treatment. E) Co‐IP assay confirmed the interaction between RPS27L and IGF1 proteins in MuSCs from M─KI mice. F) Coomassie brilliant blue staining of purified recombinant proteins (MBP‐empty, 43 KDa; MBP‐RPS27L 53 KDa; and His‐IGF1, 71 KDa; right section) and the interaction results between recombinant RPS27L and IGF1 proteins (left section). G,H) Pull‐down assays validated the direct interaction between recombinant RPS27L and **IGF1** proteins. I–K) Rescue experiments assessed proliferation activity in C2C12 myoblast transfected with: 1) si‐nc (negative control), 2) si‐Rps27l, and 3) si‐Rps27l + Igf1 overexpression (1.5 µg mL^−1^), analyzed by RT‐qPCR (**I**), western blotting (**J**), and EdU incorporation assays (**K**). L–N) High‐concentration Igf1 overexpression (1.5 µg mL^−1^) impaired the enhanced differentiation activity caused by Rps27l knockdown in C2C12 myoblast, as demonstrated by RT‐qPCR (**L**), western blotting (**M**), and MyHC immunofluorescence (**N**). The scale bars represent 100 µm. Data are presented as mean ± SEM. Exact P values are shown. Unpaired two‐tailed Student's *t*‐test (**A, C, I, J, L,** and **M**) and two‐way ANOVA (**B, D**).

Next, we identified the downstream targets of RPS27L to explore its regulatory mechanism in myogenesis. Interestingly, *Igf1*, a master regulator of myogenesis^[^ ^36^ ^]^ and muscle mass,^[^ ^37^ ^]^ exhibited an elevated expression trend in M─KI compared to WT GAS muscles (**Figure** **5**A). Meanwhile, *Igf1* mRNA exhibited significant enhanced stability in MuSCs from M─KI mice compared to WT mice following actinomycin D treatment (Figure 5B). Given that RPS27L primarily regulates its targets at post‐transcriptional level,^[^ ^38^, ^39^ ^]^ we hypothesized that RPS27L might regulate the IGF1 protein expression. Western blot analysis revealed that IGF1 protein was significantly upregulated in M─KI mice compared to their WT siblings (Figure 5C). We further treated MuSCs with the protein inhibitor cycloheximide (CHX), and found that the half‐life of IGF1 protein in M─KI MuSCs was significantly longer than that from WT MuSCs (Figure 5D). Additionally, the co‐immunoprecipitation (Co‐IP) assay in M─KI MuSCs confirmed the interaction between RPS27L and IGF1 proteins. IGF1 was robustly immunoprecipitated by an RPS27L‐specific antibody but not by the negative control IgG antibody (Figure 5E; Figure S5A, Supporting Information).

Pull‐down assays were further employed to confirm the direct interaction between IGF1 and RPS27L proteins in vitro. The recombinant proteins, including MBP (empty vector control, 43 KDa), MBP‐RPS27L (53 KDa), and His‐IGF1 (71 KDa), were purified and validated using Coomassie brilliant blue staining (Figure 5F). Results showed that IGF1 and RPS27L could be separately pulled down in a protein mixture of MBP‐RPS27L and His‐IGF1 by using different tag beads. However, no protein was detected when MBP‐RPS27L protein was replaced with its corresponding empty vector protein MBP in the protein mixture (Figure 5G,H; Figure S5B,C, Supporting Information). These results demonstrated the direct interaction between RPS27L and IGF1 in vitro. Additionally, rescue experiments revealed that *Igf1* overexpression (1.5 µg mL^−1^) in C2C12 myoblasts restored the proliferation deficit caused by Rps27l knockdown (Figure 5I–K) and attenuated the enhanced differentiation induced by Rps27l knockdown (Figure 5L–N). Furthermore, our findings indicated that Igf1 at concentrations of 1.0 and 1.5 µg mL^−1^ significantly suppressed myotube formation (Figure S5D–G, Supporting Information), consistent with previous reports indicating that Igf1 concentrations above 40 ng mL^−1^ inhibits myogenic differentiation.^[^ ^40^ ^]^ Collectively, these results demonstrate that RPS27L directly interacts with IGF1 to regulate myogenesis.

### 2.6 RPS27L Regulates Myogenesis by Liquid‐Liquid Phase Separation

**Figure 6:**
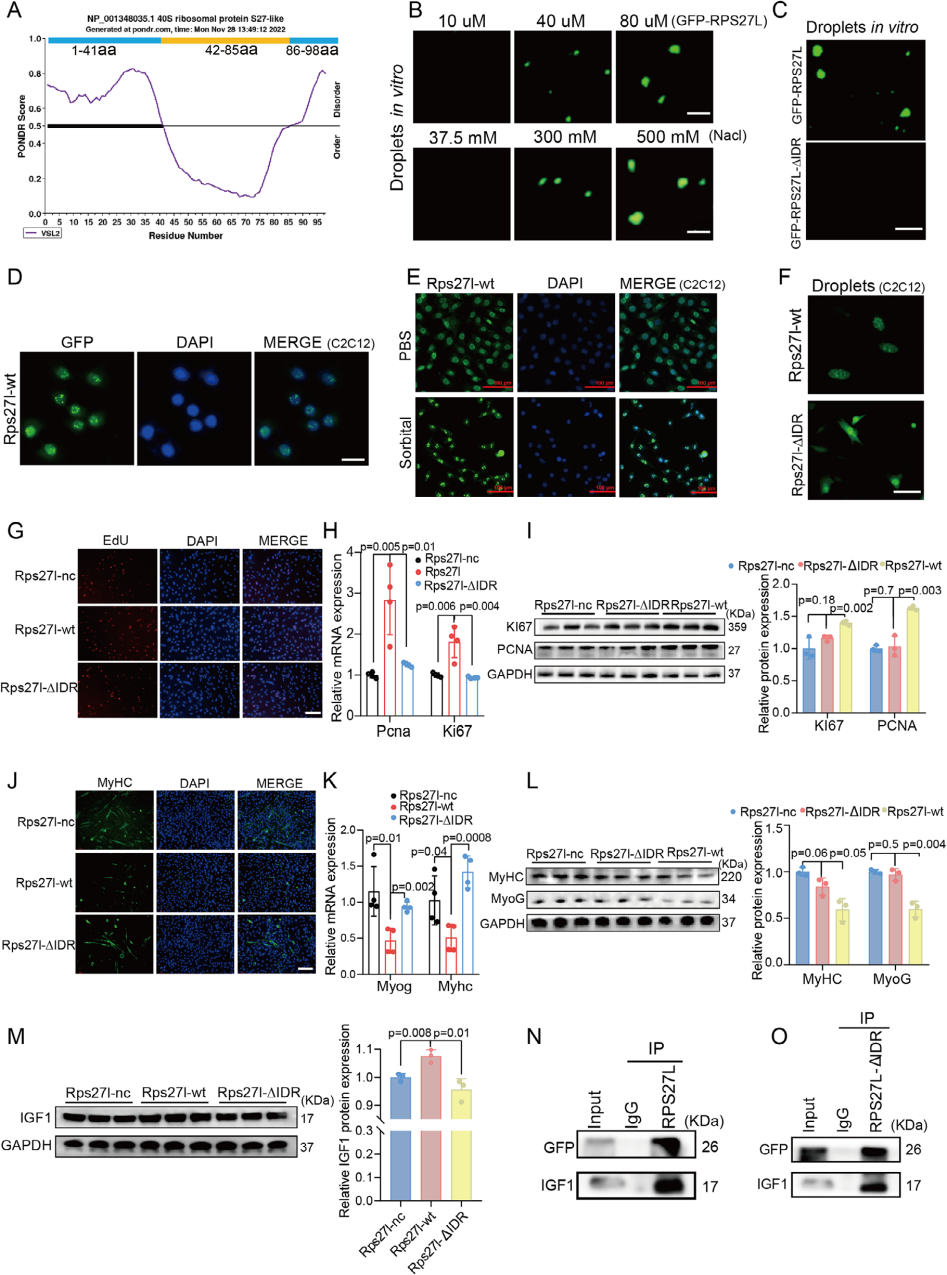
The N ‐ terminus IDR of RPS27L drives LLPS and IGF1 interaction. A) Predicting IDR regions in murine RPS27L by PONDR (http://www.pondr.com/). B) Droplets of recombinant GFP‐RPS27L were observed in vitro, exhibiting concentration and salt‐dependent. C) Droplets were observed with purified RPS27L protein in vitro, but droplet formation was abolished upon IDR deletion. D–F) Detection of RPS27L droplets in C2C12 myoblasts by immunofluorescence staining. RPS27L condensates were detected in C2C12 myoblast nuclei upon transfection with pCDNA3.1‐GFP‐Rps27l vector (Rps27l‐wt, **D**). The droplets exhibited enhanced formation (brighter and larger) under sorbitol stress compared to phosphate‐buffered saline (PBS) control **E**). In contrast, the IDR‐deletion mutant (pCDNA3.1‐GFP‐*Rps27l*‐∆IDR, abbreviated as *Rps27l*‐∆IDR, **F**) failed to form condensates. G–I) The Rps27l IDR is essential for myoblast proliferation. Rps27l overexpression enhances proliferation, this effect is markedly attenuated upon IDR deletion, as confirmed by EdU incorporation assay (**G**), alongside proliferation makers expression by RT‐qPCR (**H**) and western blot analysis (**I**). J–L) The IDR of Rps27l is dispensable for myoblast differentiation. Impaired differentiation upon Rps27l overexpression was restored following IDR deletion, as confirmed by MyHC immunostaining (**J**), aligning with the expression of myogenic differentiation markers by RT‐qPCR (**K**) and western blot (**L**). M) Western blot analysis revealed that IGF1 protein levels correlated with RPS27L expression but were significantly decreased upon deletion of the RPS27L IDR. N,O) CO‐IP assays in C2C12 myoblasts showed that IGF1 protein interacts with full‐length GFP‐Rps27l (**N**), whereas deletion of the IDR markedly reduces this interaction (**O**). The scale bars represent 100 µm. Data are presented as mean ± SEM. Exact P values are shown. Unpaired two‐tailed Student's t‐test (**H, I**, **K, L, and M**).

In light of the observation of RPS27L droplets within the nucleus of C2C12 myoblasts (Figure 4F), we predicted potential IDRs of the RPS27L protein. Two IDRs with scores greater than 0.5 were predicted by PONDR (http://www.pondr.com/) in the N‐terminus (1–41 aa) and C‐terminus (86–98 aa, **Figure** **6**A). The N‐terminus IDR was also predicted by D2P2 (https://d2p2.pro, Figure S6A, Supporting Information), suggesting that RPS27L may trigger LLPS (Figure S6B, Supporting Information) through its N‐terminus IDR.

LLPS can be driven by high concentrations of proteins containing IDRs.^[^ ^41^ ^]^ Additionally, the salt ion concentration influences LLPS by modulating electrostatic and/or hydrophobic interactions.^[^ ^42^ ^]^ To assess RPS27L‐driven LLPS in vitro, we generated two constructs using the pET‐28a vector: GFP‐Rps27l, containing full‐length Rps27l cDNA, and GFP‐Rps27l‐ΔIDR, in which the N‐terminus IDR was deleted. These constructs were expressed in *Escherichia coli* BL21 cells to obtain GFP‐RPS27L (Figure S6C, Supporting Information) and GFP‐RPS27L‐∆IDR proteins (Figure S6D, Supporting Information). Purified GFP‐RPS27L induced droplet formation, with small droplets fusing into larger ones in a salt‐dependent and concentration‐dependent manner (Figure 6B). However, no droplets were detected upon deletion of the IDR (Figure 6C), suggesting that RPS27L triggered LLPS by its IDR in vitro.

To identify whether RPS27L also triggers LLPS via its IDR in skeletal muscle cells, two additional constructs were generated: pcDNA3.1‐GFP‐*Rps27l*‐wt (Rps27l‐wt) and pcDNA3.1‐GFP‐*Rps27l*‐∆IDR (Rps27l‐∆IDR). These constructs were then transfected into C2C12 myoblasts. We observed RPS27L condensates when transfected with Rps27l‐wt by immunostaining (Figure 6D) in C2C12 myoblasts. Interestingly, droplets were larger and brighter when cells were treated with sorbitol to induce stress (Figure 6E). However, no droplets formed when the IDR of RPS27L was deleted (Figure 6F). As a complementary approach, endogenous droplets were also detected through immunostaining in M─KI MuSCs (Figure S6E, Supporting Information). Consistently, endogenous droplets in M─KI MuSCs were larger and brighter than in WT MuSCs when both were treated with sorbitol, exhibiting concentration‐dependent behavior (Figure S6F, Supporting Information). These findings demonstrated that RPS27L underwent LLPS through its IDR at the N‐terminus, both in vitro and in vivo.

To explore whether the RPS27L IDR region was functional, proliferation and differentiation assays were performed by transfecting Rps27l‐wt, Rps27l‐∆IDR and empty pCDNA3.1 vectors (Rps27l‐nc) into C2C12 myoblasts. The EdU incorporation assay demonstrated that more positive cells were detected upon overexpression of RPS27L (Rps27l‐wt) compared to the negative control group (Rps27l‐nc). However, this effect was abolished when the IDR was deleted (Rps27l‐∆IDR, Figure 6G). The increased expression of proliferation markers in the Rps27l‐wt group was not observed after IDR deletion (Figure 6H,I). Additionally, an immunostaining assay indicated a reduction in myotubes when transfected with Rps27l‐wt compared to Rps27l‐nc, but IDR deletion rescued the decreased myotube formation (Figure 6J). Concurrently, the downregulation of myogenic markers (MyoG and MyHC) by Rps27l‐wt transfection was reversed with its IDR deletion (Figure 6K,L). These results indicate the IDR is the functional region responsible for myoblast proliferation and differentiation.

Notably, the expression of IGF1 protein increased with transfection of Rps27l‐wt compared to Rps27l‐nc in C2C12 myoblast but decreased to the baseline level observed with Rps27l‐nc upon IDR deletion (Figure 6M). Furthermore, Co‐IP results revealed a markedly higher level of IGF1 protein when the full‐length CDS of RPS27L was present (Figure 6N; Figure S6G, Supporting Information), compared to the construct lacking RPS27L IDRs (Figure 6O; Figure S6H, Supporting Information). Taken together, these results indicated that RPS27L triggered LLPS via its N ‐ terminus IDR, where it interacted with IGF1 to co‐regulate myogenesis.

## 3 Discussion

**Figure 7:**
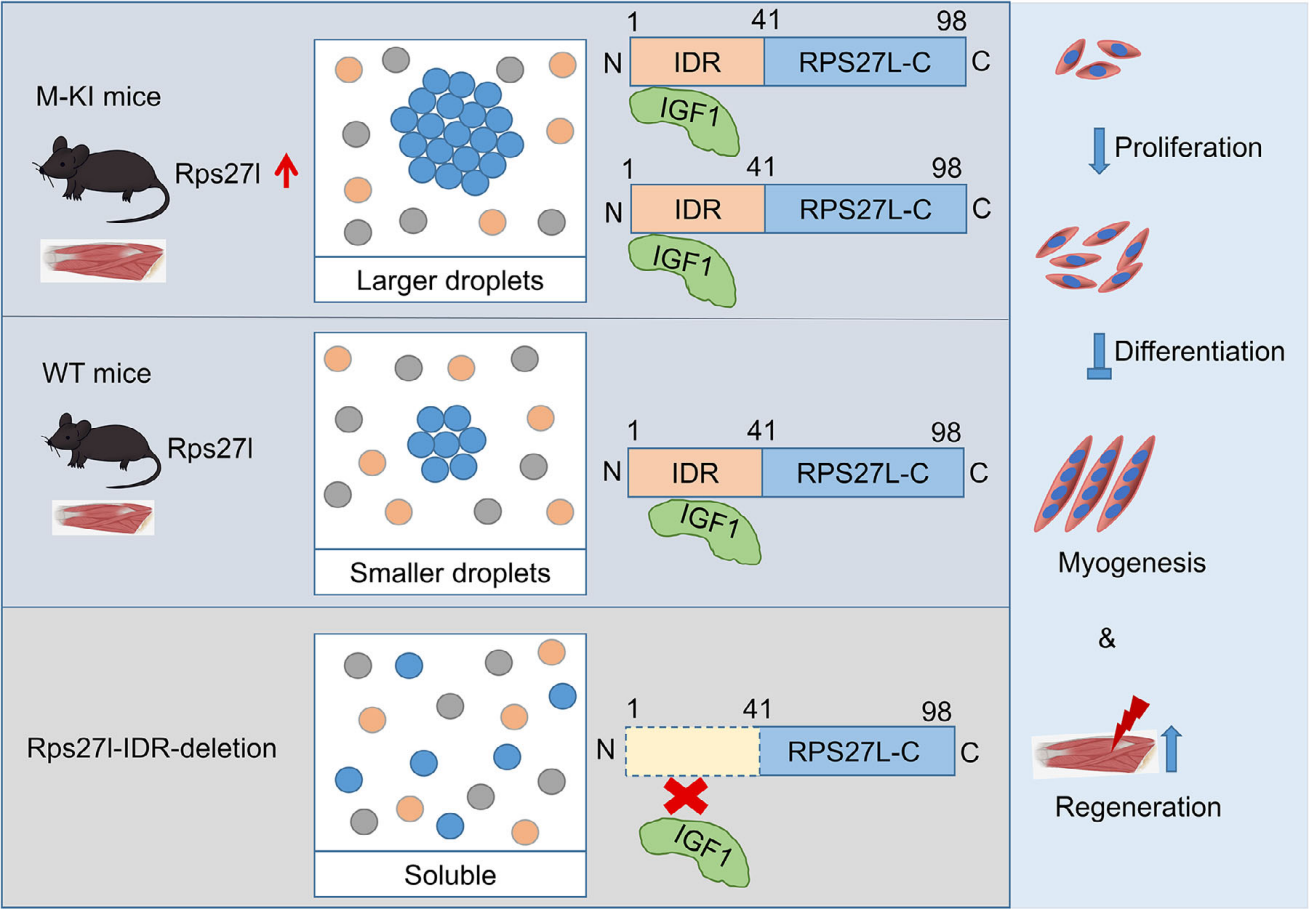
Schematic diagram of RPS27L in regulating skeletal muscle development. M─KI mice display enhanced muscle mass and body weight compared to their negative siblings (WT). Mechanistically, the IDR of Rps27l drives LLPS, evidenced by the formation of larger and brighter droplets in MuSCs from M─KI compared to WT mice. While deletion of the IDR abolishes this phase‐separation capacity. Furthermore, RPS27L directly interacts with IGF1 via its IDR, and these two proteins functionally co‐regulate myogenesis.

RBPs are key regulators in skeletal muscle development at transcriptional and post‐transcriptional levels.^[^ ^43^, ^44^ ^]^ The *Rps27l* knock‐out mice were reported to cause apoptosis and postnatal death.^[^ ^30^ ^]^ Here, we generated muscle‐specific *Rps27l* knock‐in (M─KI) mice to elucidate the functions and regulatory mechanism of RPS27L in skeletal muscle development. M─KI mice exhibited significantly elevated body weight, heavier muscle mass, and larger myofiber size. At the cellular level, we found that Rps27l promoted myoblast proliferation, migration, and fusion while suppressing myogenic differentiation. These findings underscore that gene functions identified during in vitro myogeneis may not fully recapitulate their physiological roles in vivo during skeletal muscle development and growth. This discrepancy has also been reported for other genes involved in myogenesis. For instance, the long non‐coding RNA SYISL promotes myoblast proliferation and fusion and inhibits differentiation in vitro; however, SYISL knockout mice display significantly increased muscle fiber density, muscle mass, and enhanced regenerative capacity.^[^ ^45^ ^]^ Collectively, these findings highlight the context‐dependent nature of myogenic regulation and emphasize the necessity of corroborating in vitro observations with in vivo models that recapitulate physiological conditions. Meanwhile, given that an inverse relationship exists between myofiber size and its oxidative capacity in mammals,^[^ ^46^ ^]^ results consistently demonstrated that M─KI mice exhibited an elevated percentage of fast‐glycolytic myofibers and a decreased content of slow‐twitch oxidative myofibers. These findings implied that the increased muscle mass and myofiber size in M─KI mice might be partially attributed to the remodeling of skeletal fiber types. Furthermore, M─KI mice displayed enhanced muscle regeneration ability, consistent with elevated proliferation, differentiation, migration, and fusion abilities. However, in vitro assays revealed a reduced differentiation potential, suggesting a context‐dependent regulatory mechanism. This discrepancy may arise from the absence of niche‐derived factors that are critical for regeneration in isolated culture conditions. While muscle regeneration is a multicellular process, in vitro assays are typically restricted to skeletal muscle cells alone. Hence, single‐cell multi‐omics datasets, combined with gene editing technologies applied to individual muscle fibers at various developmental and regeneration stages, will be essential to fully elucidate the dynamic expression patterns and regulatory role of Rps27l in skeletal muscle. Together, these findings underscored the critical role of RPS27L as a myogenesis regulator in skeletal muscle development and regeneration (**Figure** **7**).

SIX4 is a master TF in myogenesis. Our findings support previous reports^[^ ^47^ ^]^ that SIX4 represses myogenic gene expression and hinders myoblast proliferation. In contrast, we demonstrate that RPS27L promotes myoblast proliferation while inhibiting myogenic differentiation in murine MuSCs and C2C12 myoblasts, indicating partially opposing roles for RPS27L and SIX4 in myogenesis. We further validated co‐localization of SIX4 and RPS27L proteins in the myoblast's nuclei (Figure S6I, Supporting Information) and the direct bind of SIX4 on the RPS27L promoter, uncovering the regulation of SIX4 on RPS27L expression.

Previous reports identified several targets of RPS27L through binding at the N‐terminus, such as FANCD2, FANCI, and β‐TrCP.^[^ ^31^ ^]^ In this study, we identified IGF1, a key positive regulator of muscle mass,^[^ ^28^ ^]^ as a new target for RPS27L. The IGF1/PI3K/Akt pathway has been shown to induce hypertrophy and increase skeletal muscle protein synthesis.^[^ ^36^ ^]^ Together, our results indicated RPS27L could act as a bridge between SIX4 and IGF1, potentially activating the PI3K/Akt pathway to promote skeletal muscle growth and regeneration.

Recent studies highlighted the ability of RBPs to undergo LLPS, forming distinct condensates that support diverse cellular functions.^[^ ^27^, ^28^ ^]^ Our study elucidated that the RPS27L recruits IGF1 through LLPS to form droplets, thereby co‐regulating myogenesis. Specifically, the N‐terminal IDR of RPS27L proves to facilitate the formation of functional condensates that promote the recruitment of IGF1. This finding aligns with recent studies that highlighted how certain RBPs undergo LLPS via their IDRs. For example, the interplay between three distinct IDRs in G3BP tunes the intrinsic propensity for LLPS,^[^ ^48^ ^]^ hnRNPA1 undergoes LLPS into protein‐rich droplets mediated by a low complexity sequence domain and drives pathological fibrillization.^[^ ^28^ ^]^ Our results revealed a previously unrecognized role of RPS27L in orchestrating myogenesis through LLPS, providing anew insight into understanding the importance of LLPS in orchestrating the spatial and temporal dynamics of skeletal muscle development. However, further studies are needed to fully elucidate the role of RPS27L‐triggered LLPS in muscle biology. For example, it remains to be determined whether RPS27L‐triggered LLPS is involved in recruiting SIX4. The potential regulatory interactions between RPS27L and other key muscle regulatory factors, as well as the broader implications of LLPS in muscle health and disease, should also be explored.

In summary, we observed that muscle‐specific *Rps27l* knock‐in mice exhibit increased body weight and skeletal muscle mass, a higher proportion of glycolytic‐twitch myofibers, along with enhanced muscle regeneration capability. We uncovered the regulation of Rps27l on IGF1 expression and skeletal myogenesis through LLPS. This study highlights Rps27L as novel myogenic regulator in skeletal muscle growth and regeneration.

## 4 Experimental Section

### Ethical Statement

All animal procedures were performed according to the protocols of the Chinese Academy of Agricultural Sciences and approved by the Institutional Animal Care and Use Committee (IACUC) under the approval number AGIS‐ER‐2023‐003. Specifically, mice were randomly assigned to either treatment or control groups to ensure unbiased distribution.

### Generation of Muscle‐Specific Rps27l Knock‐in Mice

The F1 generation mice of conditional knock‐in (cKI) *Rps27l* (RosaLSL^Rps27l/+^) mice and skeletal muscle promoter‐driven Cre mice (Myf5‐cre) were generated by Shanghai Model Organisms Center, Inc. (Shanghai, China). CRISPR/Cas9 technology was used to insert the CAG‐LSL‐Rps27l‐WPRE‐pA expression box at the Rosa26 locus through homologous recombination. First, Cas9 mRNA and gRNA were obtained through in vitro transcription; then, a homologous recombination vector (donor vector) was constructed by the In‐Fusion cloning method, which includes a 3.3 kb 5′ homologous arm, CAG‐LSL‐Rps27l‐WPRE‐pA, and a 3.3 kb 3′ homologous arm. Subsequently, Cas9 mRNA, gRNA, and the donor vector were mixed and microinjected into the zygote of C57BL/6J mice to obtain F0 generation mice. After mating the F0 generation mice with C57BL/6J mice, F1 generation mice were obtained and genotyped by PCR and Sanger sequencing. The primer sequences for genotyping were listed in Table S2 (Supporting Information).

The mice were housed up to five animals per cage in the animal facilities of the Agricultural Genomics Institute at Shenzhen, Chinese Academy of Agricultural Sciences. Animals were subjected to a 12/12‐h light/dark cycle and had access to food and water ad libitum.

### Sex‐Stratified Phenotypes of Muscle‐Specific Rps27l Knock‐in Mice

Given the significantly higher body weight in male compared to female mice, all weight‐related analyses were sex‐stratified. This includes body weight growth curve, and tissue‐to‐body weight ratio for both muscular (QUA, quadriceps femoris muscle; GAS, gastrocnemius muscle; SOL, soleus muscle; TA, tibialis anterior muscle; EDL, extensor digitorum longus muscle) and non‐muscular (heart, liver, spleen, lung, kidney, stomach, pancreas, cerebellum, cerebrum and testis /ovary)organs.

### Cardiotoxin Administration

100 µL of cardiotoxin (CTX, 10 µm) (MCE, USA) was injected into the gastrocnemius muscle (GAS) of 8‐week‐old M─KI and WT mice. The mice were sacrificed to collect GAS muscles on days 3, 7, and 14 post‐CTX injury, with at least three biological replicates at each time point. In the negative control group, GAS muscles were injected with 100 µL phosphate buffer saline solution (PBS) and collected after 3 days. Samples were fixed in 4% paraformaldehyde for histology and immunohistochemistry or stored at −80 °C for RNA and protein extraction.

### Histology

Hematoxylin and eosin (H&E) staining assays were used for histology analysis. Quadriceps (QUA) and GAS muscles were paraformaldehyde‐fixed, dehydrated, and embedded in paraffin before being sectioned longitudinally into 8 µm slices and stained with H&E.^[^ ^49^ ^]^ Succinate dehydrogenase (SDH) staining was used to identify myofiber types. To do this, frozen sections (10 µm) were incubated in 0.2 m sodium phosphate buffer solution (pH 7.6) containing 0.6 mm nitro blue tetrazolium and 50 mm sodium succinate (Solarbio, China) for 30 min at 37 °C. Slides were washed with diH_2_O and mounted with aqueous mounting media. All images were visualized and captured with an Olympus BX51‐P microscope (Olympus, Japan), while Image J was used to measure myofiber sizes.

### RNA‐Sequencing Analysis

The RNA‐sequencing (RNA‐seq) libraries were prepared using the NEBNext Ultra RNA Library Prep Kit for Illumina (NEB, USA), and were sequenced on an Illumina NovaSeq platform to generate 150‐bp paired‐end reads. Three or four biological replicates were performed in each group. Following removal of reads containing adapters and poly‐N, as well as those of low quality, the clean reads were aligned with the mouse reference genome (GRCm38) using HISAT2 (version 2.0.5). The gene annotation file was downloaded from the Ensembl website (release_M25). HT‐Seq (version 0.12.4) was used to count the read numbers mapped to each gene.^[^ ^50^ ^]^ DESeq2 (version 1.22.2)^[^ ^51^ ^]^ was then applied to determine differentially expressed genes (DEGs) with a cut‐off value for FDR ≤ 0.05 and an absolute fold change ≥ 2. Gene ontology (GO) and Kyoto Encyclopedia of Genes and Genomes (KEGG) enrichment analyses were performed using the DAVID Bioinformatics Resources 6.8 (https://david.ncifcrf.gov/).^[^ ^52^ ^]^

### Cell Isolation and Culture

To isolate skeletal muscle satellite cells (MuSCs), hindlimb muscles of 1–3 days newborn mice from M─KI and WT were respectively collected and washed three times with PBS supplemented with 2% penicillin/streptomycin (PS) (Gibco, USA). Muscles were minced and digested with 0.2% type I collagenase (Sigma) for 30 min, followed by 0.25% Trypsin‐EDTA (Gibco, USA) for 20 min. Each sample was consecutively filtered through a 40‐µm cell strainer, and the cell suspension was centrifuged at 600 × g. Finally, the MuSCs were obtained by centrifugation at the bottom of the tube.

Then MuSCs were evenly pipetted and cultured in Ham's F10 nutrient medium (Gibco, USA) supplemented with 20% FBS (Gibco, USA), 5 ng mL^−1^ bFGF, and 1% PS. HEK293T and C2C12 myoblasts were cultured in Dulbecco's modified eagle medium (DMEM, Gibco, USA) supplemented with 10% FBS and 1% PS in a humidified incubator with 5% CO2 at 37 °C. When MuSCs and C2C12 myoblasts reached 80%–90% confluency, the growth medium was replaced with differentiation medium containing 2% heat‐inactivated horse serum (BI, USA) and 1% PS.

### Plasmid Construction, RNA Interference, and Transfection

To construct overexpression vectors, the full‐length cDNA sequence of mouse Rps27l, Six4, and Igf1 were cloned into the pcDNA3.1 vector (Genecreate Biotech, China), respectively, and a mock vector lacking the corresponding sequence served as a negative control. To knock down Rps27l, three siRNAs (si‐Rps27l) and a negative control (siRNA‐NC) were synthesized (RiboBio Biotech, China), and the siRNA with the highest knock‐down efficiency (≈65%) was used for further analysis. The siRNA sequences were listed in Table S2 (Supporting Information).

To construct promoter luciferase reporter plasmids, five continuous regions (P1, +318 bp to 506 bp; P2, +318 bp to −506 bp; P3, +318 bp to −1006 bp; P4, +318 bp to −1506 bp; P5, 318 bp to −2006 bp) of the Rps27l promoter were subcloned into the pGL3‐basic vector (Genecreate Biotech, China) using the Sac1/XhoI sites. Transient transfection was performed using Lipofectamine 3000 according to the manufacturer's instructions (Invitrogen, USA).

### Luciferase Reporter Assay

The core promoter region of Rps27l was identified by co‐transfecting Rps27l promoter luciferase reporter vectors (pGL3‐P1 to ‐P5) and the pGL3‐basic empty vector with pRL‐TK plasmids in HEK293T cells. To validate the interaction between Six4 and Rps27l, potential binding sites of the core promoter were mutated. The Six4 overexpression vector was co‐transfected with wild‐type and mutant‐type core promoter regions in HEK293T cells and C2C12 myoblasts, while the group co‐transfected with pGL3‐Basic and empty pcDNA3.1 vectors served as a negative control. Luciferase assays were performed 48 h after transfection using the Dual Luciferase Reporter Assay System (Promega, USA). Firefly luciferase activity was normalized to Renilla luciferase activity for each transfected well.

### RNA Preparation and RT‐qPCR

Total RNAs were extracted from tissues and cells using TRIzol reagent (TaKaRa, Japan), before RNAs were reverse transcribed into cDNAs using the HiScript III 1st Strand cDNA Synthesis Kit (+gDNA wiper) according to the manufacturer's instructions (Vazyme, China). Real‐time quantitative PCR (RT‐qPCR) was performed using the ChamQ SYBR qPCR Master Mix (Without ROX) (Vazyme, China). RT‐qPCR data were analyzed using the ΔΔCt method and normalized to *Gapdh* or 18s RNA. Primer sequences were listed in Table S2 (Supporting Information).

### Cell Proliferation and Cell Cycle Assay

For cell proliferation analyses, 10 µL Cell Counting Kit‐8 (CCK‐8) reagent (Beyotime, China) was added to each well of a 96‐well plate containing cells (C2C12 myoblast cells and MuSCs) and incubated at 37 °C for 45 min. The absorbance of cells at 450 nm was measured using a microplate reader (Tecanspark, Switzerland) at 0, 24, 36, 48, and 72 h after transfection. Five independent replicates were performed for each group.

The Ethynyl‐2′‐deoxyuridine (EdU) assay, another method for analyzing cell proliferation, was performed using Cell‐Light EdU Cell Proliferation Kit (Beyotime, China). Cells were seeded in a 12‐well plate and transfected when they reached 50% confluence. After cultivation with growth medium for 24 h, cells were incubated in 50 µm of EdU for 1 h, fixed with 1 mL of 4% paraformaldehyde for 15–30 min, and permeabilized with 0.5% Triton X‐100 for 15 min. The click reaction mixture was then prepared and added to each well. After incubation at room temperature in the dark for 30 min, cells were stained with DAPI at a dilution of 1:1000 for nuclear staining to count the whole cells. The number of EdU‐stained cells was counted using a fluorescence microscope for the proliferating cells.

Flow cytometry analysis of the cell cycle was then performed using a BD Accuri C6 flow cytometer (BD Biosciences, USA) as previously described,^[^ ^53^ ^]^ and data were processed using the ModFit LT 5.0 software (Verity Software House, USA).

### Immunohistochemistry and Immunostaining

Cells or tissue sections were fixed with 4% paraformaldehyde for 20 min at room temperature, washed with PBS, and permeabilized with 0.5% Triton‐100 for an additional 10 min. For labeling, the cells/sections were then incubated with 5% bovine serum albumin (BSA) at room temperature for 1 h, followed by overnight incubation with primary antibodies (diluted in 5% goat PBS) at 4 °C, before washing in PBS three times and incubation with secondary antibodies for 1 h at room temperature. DAPI dilution solution (1:1000) was then used to stain cell nuclei. The primary and secondary antibodies used include anti‐Myhc (1:400, DSHB, USA), anti‐Myh7, anti‐Myh4 (1:400, sigma, USA), anti‐eMHC (1:400, DSHB, USA), and anti‐Laminin (1:400, abcam, USA). Alexa Fluor 594 goat anti‐mouse IgG (1:400, Proteintech) and Alexa Fluor 488 goat anti‐rabbit/anti‐mouse IgG (1:1000, abcam). The labeled cells were observed under a fluorescence microscope, and the cell area labeled by the antibody was measured using Image J software.

### Transwell Assay

The transwell assay was employed to detect cell migration. 200 µL of culture medium (DMEM + 1% FBS+1% PS; Gibco, USA) and 500 µL of growth medium (DMEM + 10% FBS+ 1% PS; Gibco, USA) were added to the upper and lower chambers of the Transwell (Corning, USA), respectively to induce cell migration. MuSCs at a concentration of 3 × 10^5^ mL^−1^ were added to the upper chamber and incubated in a cell culture incubator for 24‐36 h. Non‐invading cells were removed from the upper surface of the membrane with a cotton swab. The invading cells were then fixed with 4% paraformaldehyde for 10 min, washed with PBS, stained with DAPI for 10 min at room temperature, and subsequently washed with PBS three times. Stained cells were observed and counted under a microscope (10X). At least three fields of view were observed for each sample.

### Blood Glucose Measurements

Total 5 µL of blood collected from the tail vein was dropped onto a glucose test strip (Accu‐Check Active, Roche) and measured by a glucometer (Accu‐Check Active, Roche). For glucose tolerance tests (GTT), mice were given intraperitoneal injection (I.P.) of 30% glucose (a dose of 1 g kg^−1^ of body weight) after overnight fasting, and tail blood glucose concentrations were monitored in 0, 15, 30, 60, 90, and 120 min after glucose administration. For insulin tolerance tests (ITT), mice were fasted for 6 h before I.P. administration of human insulin (Santa Cruz) (0.75 U kg^−1^ of body weight), and tail blood glucose concentrations were monitored as GTT timetable. For both GTT and ITT, each mouse was singly caged with blinded cage numbers and random orders.

### Western Blotting

Total protein was extracted from tissues or cells according to reducing agent and protease Inhibitor buffer (RAPI) buffer method as previously described. Proteins were separated by SDS‐polyacrylamide gel electrophoresis (SDS‐PAGE) and transferred to nitrocellulose membranes. The membranes were blocked with 5% skim milk for 2 h at room temperature and subsequently probed with primary antibodies overnight at 4 °C. The antibodies used at a 1:1000 dilution include: PCNA (Abcam, ab18197, USA), CyclinA2 (Abcam, ab137769, USA), Ki67 (Novus, NB500‐170, USA), Myog (Affinity, DF8273, China), Pax3 (Proteintech, 21386‐1‐AP, China), Mymk (Abclonal, A18158, China), Myh7 (DSHB, BA‐D5, USA), Myh4 (DSHB, 10F5, USA), eMHC (DSHB, BF‐G6, USA), Igf1 (Abcam, ab223567, USA), Gapdh (CST, 2118S, USA), β‐actin (Proteintech, 20536‐1‐AP, China) and Rps27l (Invitrogen, 13‐1600, USA). The membranes were washed with PBS‐Tween and incubated for 1 h with horseradish peroxidase‐conjugated secondary antibodies (1:1000, Proteintech). Protein bands were detected after treatment with SuperSignal West Femto agent (Thermo Scientific, USA).

### Co‐Immunoprecipitation

Co‐immunoprecipitation (Co‐IP) was performed using a Co‐IP Assay Kit (Thermo Scientific, USA). First, the protein lysate (500–1000 µg) of each sample was mixed with 2–10 µg immunoprecipitation antibody, then the mixture was incubated overnight at 4 °C to form immune complexes, before the mixture was changed to a centrifuge tube containing magnetic beads and incubated at room temperature for 1 h to form beads‐immune complexes. The magnetic beads were collected using a magnetic holder to remove unbound samples. The beads were then washed using 500 µL immunoprecipitation lysis buffer three times. Finally, the binding protein complexes were obtained using 100 µL eluent and examined by western blotting.

### Chromatin Immunoprecipitation

Chromatin immunoprecipitation (ChIP) was initially conducted through a crosslinking reaction, which was subsequently terminated using glycine in C2C12 cells treated with 1% formaldehyde. Samples were then lysed on ice for 10 min before mechanical chromatin shearing was performed by sonicating for 10 s and resting 10 s in one cycle, and repeating for a total of 10 cycles. The anti‐Six4 antibody (Santa Cruz, USA) was added to form the antibody‐target protein‐DNA complex. Protein A‐Sepharose beads were used to immunoprecipitated the complex. After washing and reversing the crosslinking, the enriched DNAs were purified and examined by RT‐qPCR. The primer sequences were listed in Table S2 (Supporting Information).

### Protein Expression and Purification

The fusion proteins (His‐IGF1 and MBP‐RPS27L) were expressed in *Escherichia coli* BL21. Briefly, cultures were grown to an OD_600_ of 0.6 and then induced with 0.5 mm isopropyl‐β‐D‐thiogalactoside with the optimum culture temperature and sonicating time (His‐IGF1 protein was premium performed in 16 °C and sonicating for 30 min, whereas MBP‐RPS27L was premium performed in 28 °C with the same sonication duration). Cells were then harvested by centrifugation, washed twice with 30 mL of suspension buffer (15 mm Tris‐HCl (pH 6.5), 0.5 M NaCl, and 10% glycerol), and resuspended in 50 mL of suspension buffer. The suspensions were disrupted using an ultrasonic cell crusher JY92‐IIN (Scientz, China) with 10‐s bursts and 10‐s rest periods until the suspensions were cleared and then centrifuged at 17000 rpm for 40 min at 4 °C. The tagged proteins were purified using corresponding beads. Purified recombinant proteins were concentrated using Amicon ultra‐15 concentrators (Millipore, USA) and equilibrated in storage buffer (40 mm Tris‐HCl (pH 7.2), 500 mm KCl, 0.2 mm EDTA, 0.2 mm DTT, and 50% glycerol) at −80 °C. Protein concentrations were determined using a Genova Nano instrument (Bibby Jenway, UK).

### Coomassie Brilliant Blue Staining

Coomassie brilliant blue staining was used to detect the expression of fusion proteins. The purified proteins (MBP protein, MBP‐RPS27L, and His‐IGF1) were denatured after dilution. Gel electrophoresis was performed by SDS‐PAGE, and then Coomassie brilliant blue R‐250 solution (2X Solution, Sangon Biotech, China) was used for staining at room temperature for 1 h. Pictures were taken after water decolorization overnight.

### Pull‐Down Assay

Proteins of MBP (43 KDa), MBP‐RPS27L (53 KDa), and His‐IGF1 (71 KDa) were expressed in *Escherichia coli* BL21 and purified according to standard protocols.^[^ ^54^ ^]^ Aliquots of beads (100 µL beads containing ≈15 ug of protein) with MBP or MBP‐RPS27L proteins were incubated for 2 h at 4 °C with His‐IGF1 protein. After being washed with PBS buffer, bound proteins were eluted from beads with 50 uL of elution buffer (20 mm reduced glutathione in 50 mm Tris‐Cl, pH 8.0), resolved on a 12.5% SDS‐PAGE gel, and then immunoblotted with anti‐His or anti‐MBP antibodies at a 1:1000 dilution. The primer sequences were listed in Table S2 (Supporting Information).

### Electrophoretic Mobility Shift Assays (EMSAs)

Two 50 bp DNA probes, wild‐type and mutant‐type, were synthesized based on the binding sites (GGTCTGGTTTC) between the Rps27l promoter and Six4. The wild‐type probe was labeled with biotin at the 5′ end using the EMSA Probe Biotin Labeling Kit (Beyotime, China) and purified using the phenol‐chloroform extraction method. The sequences of DNA oligos were provided in Table S2 (Supporting Information). Labeled DNA probe (1 µm) and 0.5–4 µg of His‐SIX4 protein was mixed in 10 µL reactions using the Chemiluminescent EMSA Kit (Beyotime, China). Mixtures were incubated at 25 °C for 20 min and then separated by 4% non‐denaturing polyacrylamide gel electrophoresis at 100 V in 0.5 × Tris‐borate‐EDTA buffer (pH 8.0). In competition analyses, unlabeled (cold and mutant) probes were added before the addition of labeled probes. The gels were incubated with a streptavidin‐HRP conjugate, and chemiluminescence was measured using Image LabTM (BIO‐RAD ChemiDocTM XRS+, USA).

### Droplet Formation In Vitro

To assess the dose‐dependent formation of RPS27L droplets in vitro, purified RPS27L fusion was diluted to specified concentrations (10, 40, and 80 µm). To evaluate the salt ion dependence of droplet formation, 35 µm RPS27L was mixed with different salt concentrations (37.5, 300, and 500 mm). Each 5 µL of diluted protein solution was then added to a slide, where droplet formation was observed and photographed by a laser confocal microscope. Image J software was used to measure the number and size and of droplets. Results were quantitated based on three sizes of 16 × 124 µm^2^.

### Statistics and Reproducibility

The data were presented as mean ± SEM. The number of independent biological replicates per experiment is shown in figure legends. For both murine and cellular studies, no data was excluded from the analysis. Test and control groups were randomly selected to ensure an unbiased experimental design. Statistical analysis was performed using Prism 7 software (GraphPad Software, San Diego, CA, USA). Unpaired two‐tailed Student's *t*‐test and two‐way ANOVA were used to analyze the statistical significance. Exact *P* values were reported. Differences in means were considered statistically significant when they reached *P* < 0.05. Significance levels were indicated as follows: ^*^ *P* < 0.05, ^**^ *P* < 0.01, and ^***^ *P* < 0.001.

## Conflict of Interest

The authors declare no conflict of interest.

## Author Contributions

Z.L.T. and Y.L.Y. conceptualized the project. X.Q.L., Y.L.Y. and Z.L.T. designed experiments. X.Q.L., Y.L.Y., J.Y.Y., J.J.L., Y.W.L., S.Y.Y., and L.J.C. performed molecular, cellular, and other experiments. M.Z. and X.H.F. administered RNA‐seq data analyses. R.P.C. provided some useful websites for TFs prediction. X.Q.L., Y.J.T., and Y.X.H. raised the mice. X.Q.L. wrote the manuscript. Z.L.T., Y.L.Y., G.G., and H.C. revised the paper. All authors approved the final version of the manuscript.

## Supporting information



Supporting Information

Supporting Information

Supporting Information

## Data Availability

The RNA‐seq data has been deposited in the China National GeneBank Database (CNGBdb) under accession number CNP0004843 (https://db.cngb.org/search/project/CNP0004843/).
