# Supplementary material for: RPS27L Enhances Myogenesis and Muscle Mass by Targeting IGF1 Through Liquid‐Liquid Phase Separation
Source: Adv Sci (Weinh). 2025 Aug 31;12(44):e12354. doi: 10.1002/advs.202512354 (PMC12667544; doi:10.1002/advs.202512354)
Supplement: Supplementary file 1 — Supporting Information [file ADVS-12-e12354-s002.docx]

**Supplementary Figures:**


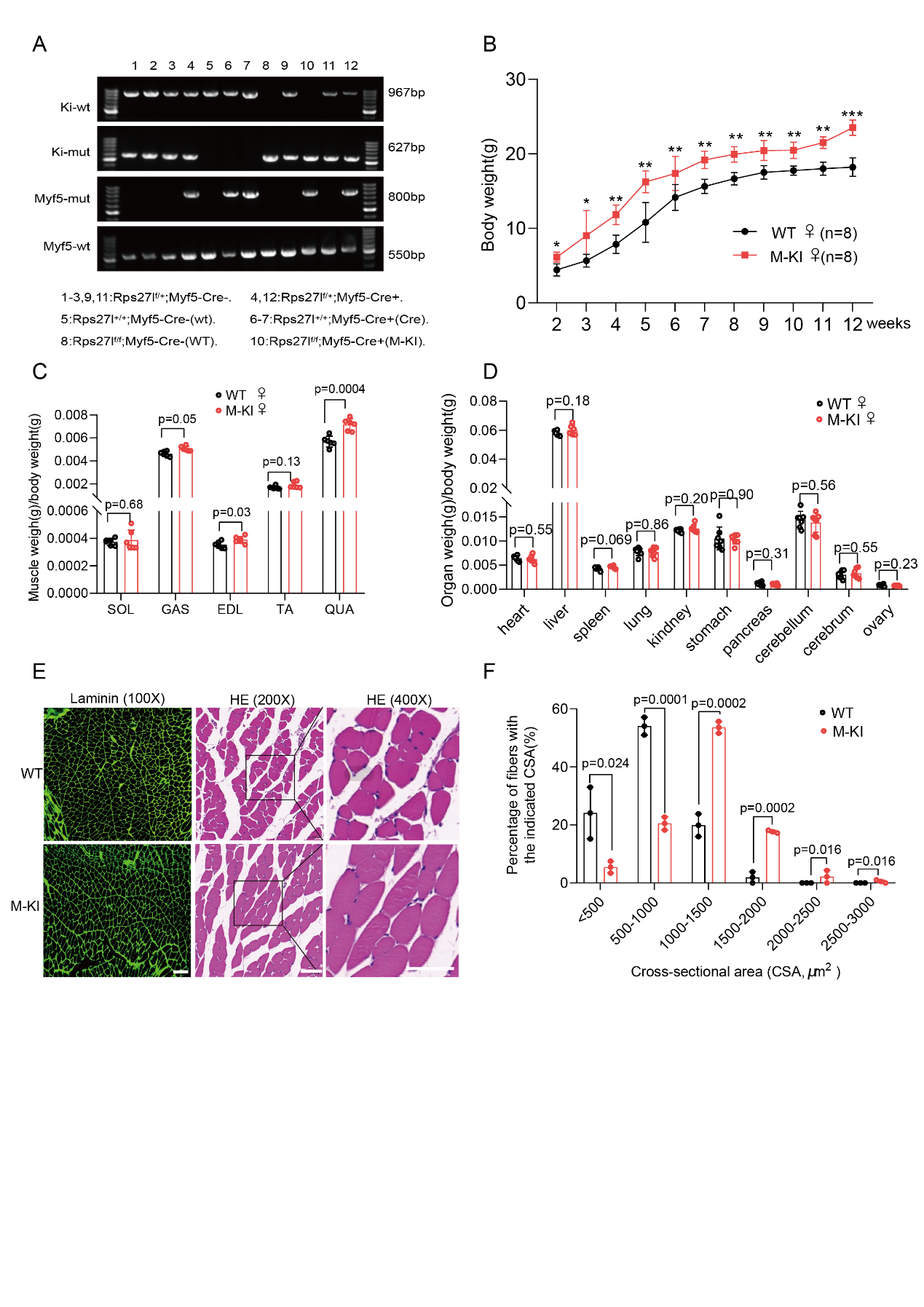


**Figure S1. Female M-KI mice showed heavier body weight and muscle mass. Related to Figure 1.** **A**) Genotyping of muscle specific *Rps27l* conditional knockin mice. **B**) Growth curves comparing body weights of female M-KI and WT mice from 2 to 12 weeks postnatal. **C**,**D**) Ratio of five typical muscles (**C**) and other non-muscle organs (**D**) to body weight of female mice (n≥ 6 individuals per group). QUA, quadriceps femoris muscle; GAS, gastrocnemius muscle; SOL, soleus muscle; TA, tibialis anterior muscle; EDL, extensor digitorum longus muscle. **E**,**F**) Representative laminin immunofluorescence (left) and H&E-stained sections (200×, 400×) of GAS muscles (**E**). Data analysis revealed a right-shifted myofiber size distribution in M-KI mice compared to WT controls (**F**). The scale bars represent 100 μm. Data are presented as mean ± SEM. Exact P values are shown, *P < 0.05, **P < 0.01 and ***P < 0.001. Unpaired two-tailed Student’s t-test (C, D and F) and two-way ANOVA (B).


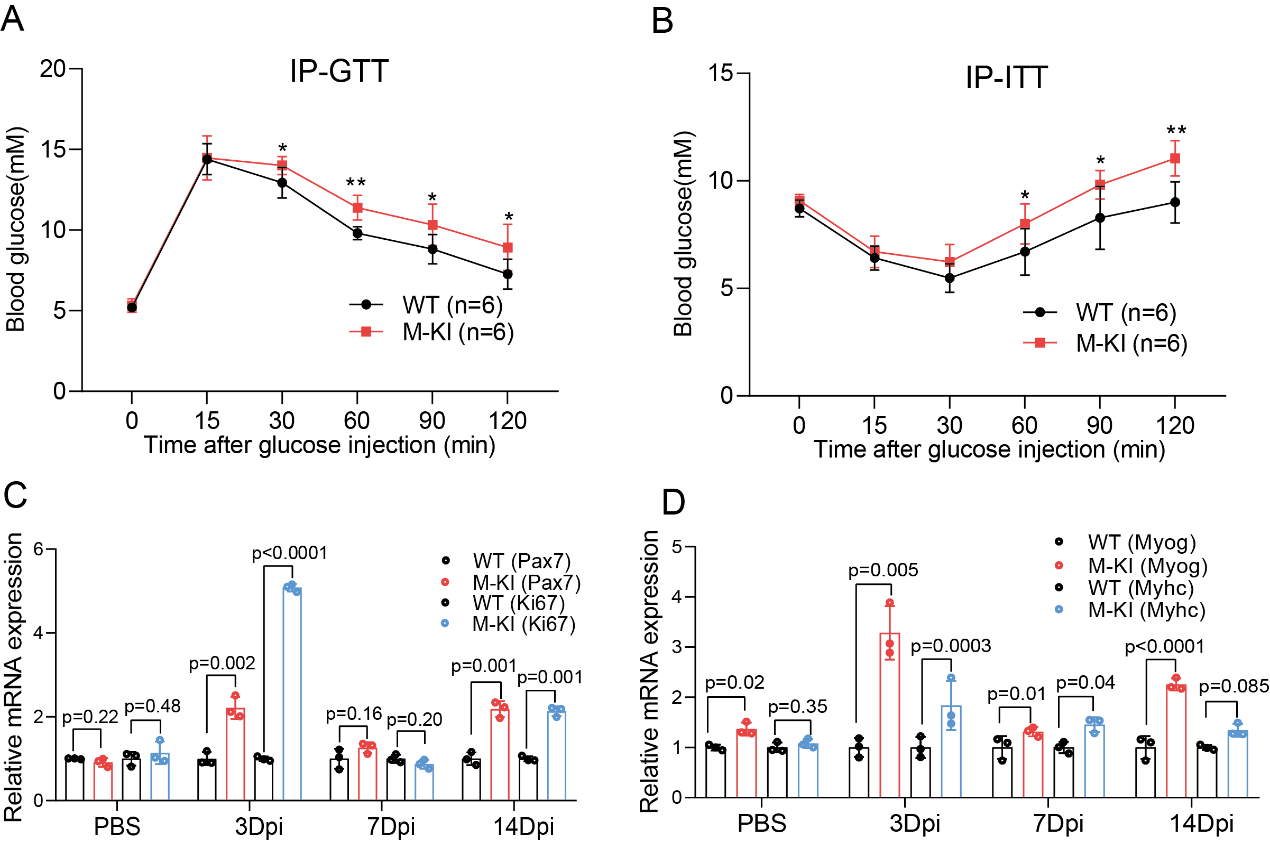


**Figure S2. M-KI mice exhibit increased insulin resistance, reduced glucose tolerance and enhanced muscle regeneration capability. Related to Figure 2.** **A**,**B**) Blood glucose levels during IP-GTT (**A**) and IP-ITT (**B**) in M-KI and WT mice at the age of 2 months, n=6 per group. **C**,**D**) Expression of proliferation (**C**) and differentiation (**D**) markers was quantitated by RT-qPCR at multiple regeneration timepoints in GAS muscle from M-KI and WT mice. Data are presented as mean ± SEM. Exact P values are shown, *P < 0.05, **P < 0.01 and ***P < 0.001. Unpaired two-tailed Student’s t-test (**C**, **D**) and two-way ANOVA (**A**, **B**).


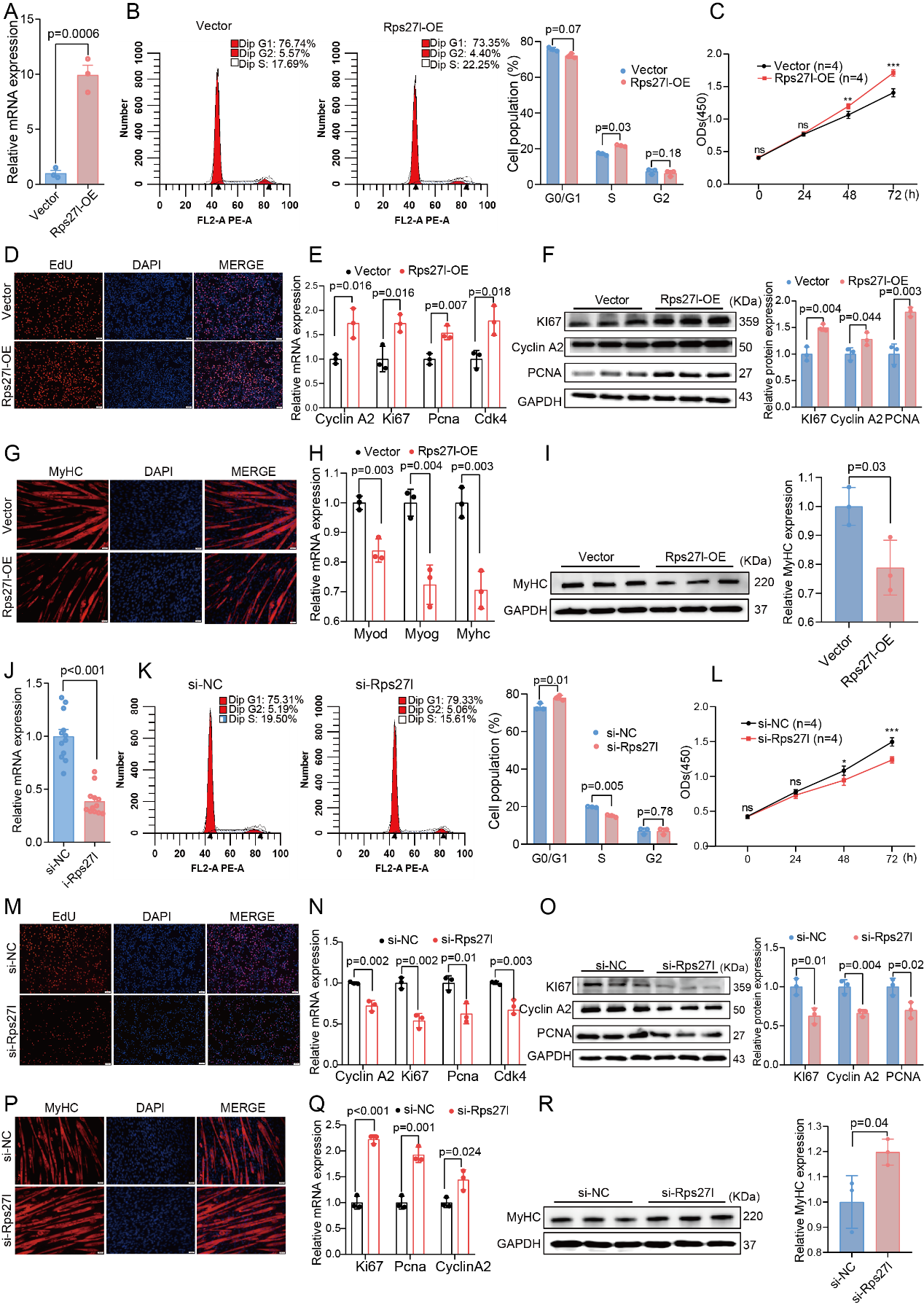


**Figure S3. RPS27L promotes myoblast proliferation and represses differentiation in C2C12 myoblasts. Related to Figure 3.** (**A-J**) The assessment of RPS27L on myoblast proliferation and differentiation with *Rps27l* overexpression. *Rps27l* expression was significantly higher in Rps27l-OE transfection compared to empty vector controls (**A**). Flow cytometric analysis revealed a significant increase in S-phase population in Rps27l-OE transfected C2C12 myoblasts compared to empty vector controls (**B**). **C,D**) The proliferative capacity of *Rps27l* overexpression was significantly enhanced in C2C12 myoblasts, as evidenced by CCK-8 (**C**) and EdU incorporation assays (**D**). EdU staining (red) for positive cells; DAPI staining (blue) for cell nuclei. **E**,**F**) The increased proliferative capacity of *Rps27l* overexpression was evaluated through expression analysis of proliferation markers by RT-qPCR (mRNA, **E**) and western blotting (protein, **F**). (**G-I**) The decreased differentiation ability of *Rps27l* overexpression was assessed through expression analysis of relative markers by immunofluorescence staining (**G**), RT-qPCR (mRNA, **H**) and western blotting (protein, **I**). (**J-R**) The functional role of RPS27L in myoblast proliferation and differentiation was assessed through siRNA-mediated knockdown. The knockdown efficiency of *Rps27l* by siRNA in C2C12 myoblasts was assessed by RT-qPCR (**J**). *Rps27l* knockdown significantly reduced the S-phase fraction by flow cytometry (**K**). **L,M**) *Rps27l* knockdown significantly decreased the proliferation capacity, as evidenced by the results of CCK-8 assay (**L**) and EdU incorporation assays (**M**). **N**,**O**) *Rps27l* knockdown significantly reduced proliferation markers at both mRNA (RT-qPCR, **N**) and protein (Western blot, **O**) levels. (**P-R**) *Rps27l* knockdown significantly enhanced differentiation markers at both mRNA and protein levels, as demonstrated by immunofluorescence staining (**P**), RT-qPCR (**Q**) and western blot analysis (**R**). The scale bars represent 100 μm. Data are presented as mean ± SEM. Exact P values are shown, *P < 0.05, **P < 0.01 and ***P < 0.001. Unpaired two-tailed Student’s t-test (**A**, **B**, **E**, **F**, **H**, **I**, **J**, **N**, **O**, **Q**, **R**) and two-way ANOVA (**C**, **L**).


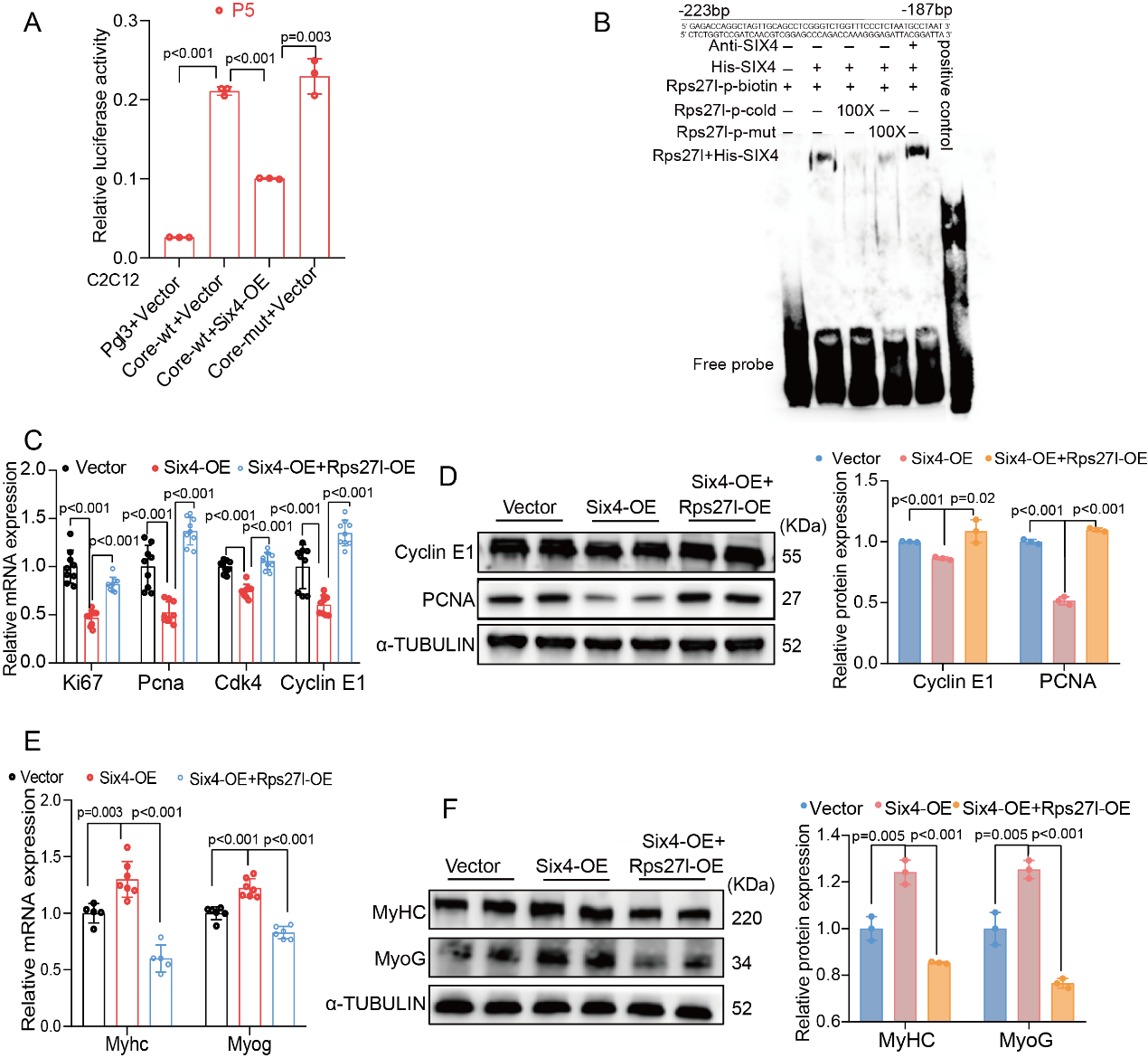


**Figure S4. Rps27l overexpression rescued the inhibited proliferation and promoted differentiation abilities of Six4 in C2C12 myoblasts. Related to Figure 4. A)** *Six4* overexpression significantly repressed the dual-luciferase activity driven by the full length *Rps27l* promoter (P5) in C2C12 myoblasts. **B**) The binding interaction between murine SIX4 and Rps27l promoter was confirmed by replicating EMSA assay *in vitro*. (**C**-**D**) The reduced proliferation ability by *Six4* overexpression was rescued by *Rps27l* overexpression, as evidenced by RT-qPCR (mRNA, **C**) and Western blot analysis (**D**) in C2C12 myoblasts. (**E**-**F**) *Rps27l* overexpression abolished the enhanced differentiation capacity of *Six4* overexpression, as demonstrated by RT-qPCR (mRNA, **E**) and Western blot analysis (**F**) in C2C12 myoblasts. The scale bars represent 100 μm. Data are presented as mean ± SEM. Exact P values are shown. Unpaired two-tailed Student’s t-test (**A**, **C**, **D, E** and **F**).


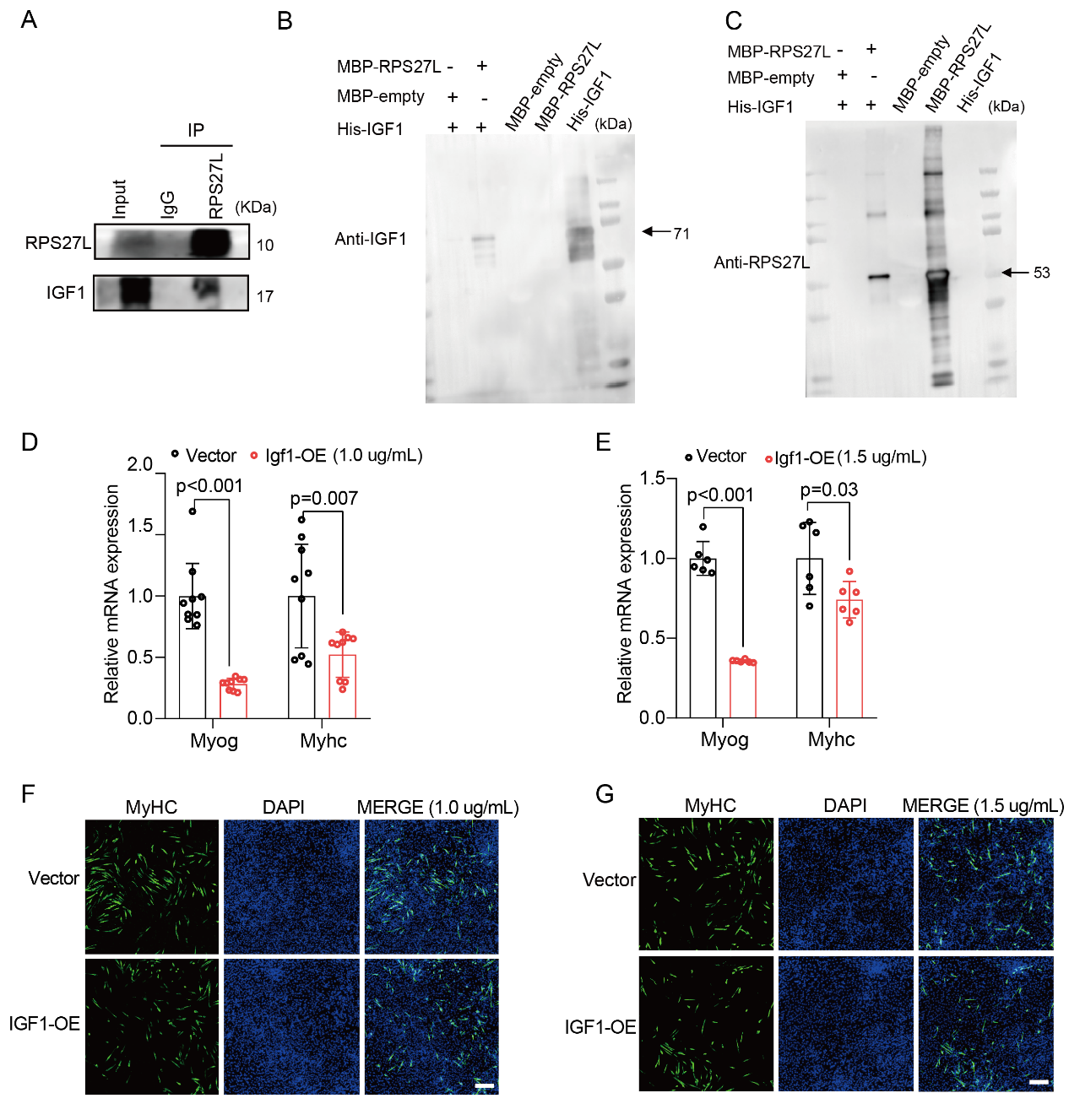


**Figure S5. High concentration of IGF1 inhibits myoblasts differentiation. Related to Figure 5.** **A**) The interaction between RPS27L and IGF1 was validated by CO-IP assay in MuSCs from M-KI mice, with replicate experiments confirming reproducibility. **B,C**) The RPS27L-IGF1 interaction was confirmed through reproducible *in vitro* pull-down assays using purified recombinant proteins. (**D-G**) IGF1 at concentrations of 1.0 and 1.5 μg/mL significantly inhibited myoblast differentiation, as evidenced by downregulated myogenic markers in both RT-qPCR (**D and E**) and immunofluorescence staining (**F** and **G**). The scale bars represent 100 μm. Data are presented as mean ± SEM. Exact P values are shown. Unpaired two-tailed Student’s t-test (**D** and **E**).


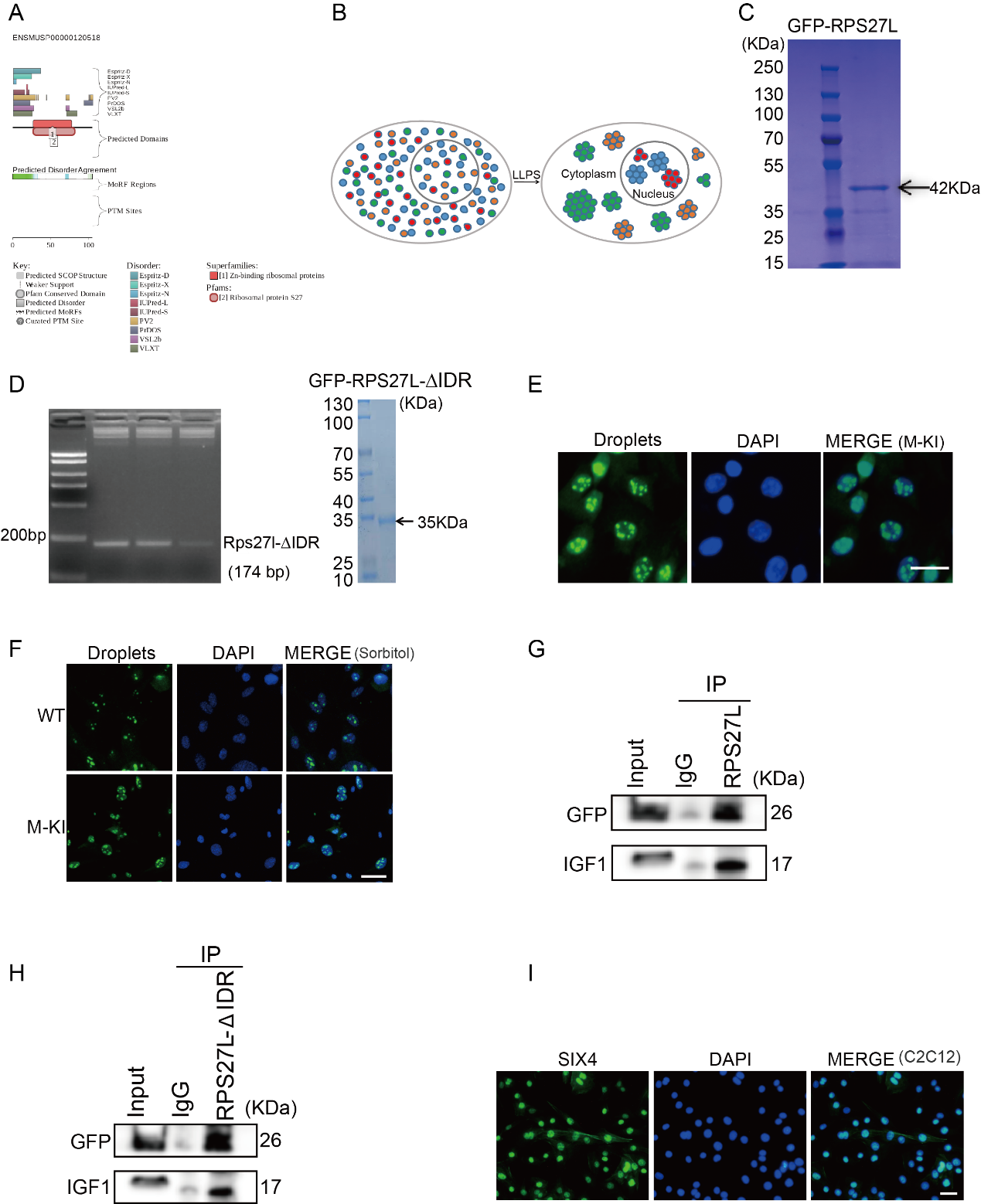


**Figure S6. RPS27L regulates myogenesis by SIX4/RPS27L/IGF1 axis. Related to Figure 6. A**) Prediction of IDRs in RPS27L (<https://d2p2.pro>). **B**) Pattern diagram illustrates the occurrence of liquid-liquid phase separation (LLPS). **C**) Coomassie brilliant blue staining to confirm the purified GFP-RPS27L. **D**) Successful generation of the GFP-RPS27L-ΔIDR construct was validated by agarose electrophoresis at DNA level and Coomassie staining at protein level. **E**) Droplets were observed in M-KI MuSCs via RPS27L antibody immunostaining. **F**) MuSCs from M-KI exhibited larger and brighter condensates than WT controls following sorbitol treatment (0.5 M, 1h). **G**,**H**) Replicant Co-IP assays confirmed that RPS27L interacts with -IGF1 via its IDR, as evidenced by significant decreased IGF1 pulldown following IDR deletion. **I**) Immunofluorescence analysis revealed predominant nuclear localization of SIX4 (green), DAPI (blue). The scale bars represent 100 μm.
